# Age-dependent vulnerability to spatial memory interference in APP/PS1 mice

**DOI:** 10.3389/fnagi.2026.1794153

**Published:** 2026-06-02

**Authors:** Dejana Mitrovic Tartanoglu, Can Hicabi Tartanoglu, Charlotte Sonnenberg, Marta Sabariego, Matthias G. Haberl, Silvia Viana da Silva

**Affiliations:** 1 German Center for Neurodegenerative Diseases (DZNE), Berlin, Germany; 2 MedNeuro Program, Charité Universitätsmedizin Berlin, Berlin, Germany; 3 Neuroscience and Behavior, Mount Holyoke College, South Hadley, MA, United States; 4 Institute of Neuroanatomy, Charité Universitätsmedizin Berlin, Corporate Member of Freie Universität Berlin, Humboldt-Universität Berlin, and Berlin Institute of Health, Berlin, Germany; 5 Bernstein Center for Computational Neuroscience (BCCN), Humboldt-Universität zu Berlin, Berlin, Germany; 6 Einstein Center for Neurosciences (ECN), Berlin, Germany

**Keywords:** aging, Alzheimer’s disease, APP/PS1 AD mice, behavior, c-Fos, hippocampus, memory updating, spatial memory

## Abstract

**Background:**

While Alzheimer’s disease (AD) is well known for progressive memory impairment, less is understood about how amyloid pathology affects flexible updating of competing spatial representations. Here, we used the Objects in Updated Locations (OUL) paradigm to investigate how amyloidosis influences spatial memory updating and the handling of competing mnemonic information in APP/PS1 mice.

**Methods:**

APP/PS1 and wild-type control mice were tested in the OUL paradigm, which probes memory for object displacement and subsequent updating of overlapping spatial representations. Exploration behavior was quantified using an automated behavioral tracking pipeline based on object proximity and gaze orientation criteria and validated against blinded manual scoring. Object discrimination performance was assessed during the object displacement session and during the subsequent updating performance session involving competing spatial information. cFos immunohistochemistry following novelty exposure was used to assess neuronal recruitment associated with memory performance.

**Results:**

During the object displacement session, control mice reliably discriminated the displaced object location, whereas APP/PS1 mice showed weaker and more variable performance, with a greater proportion failing to reach discrimination criterion levels. During the subsequent updating performance session, control mice demonstrated more flexible adaptation across overlapping spatial representations, while APP/PS1 mice exhibited weaker performance under conditions involving competing spatial traces. In APP/PS1 mice, longer exploration latencies were negatively associated with discrimination performance specifically under competing-memory conditions. Age-stratified analyses revealed a significant genotype effect in older animals, driven primarily by the condition involving overlapping spatial representations. In control mice, cFos expression in hippocampal regions associated with spatial memory processing correlated with behavioral performance, whereas these relationships were absent in APP/PS1 mice.

**Conclusion:**

These findings indicate that amyloidosis is associated with reduced reliability of spatial memory performance and impaired handling of competing spatial information, particularly under conditions requiring flexible updating of overlapping mnemonic representations. The results further suggest increased vulnerability to interference-related spatial memory deficits with age. Together, these findings support the utility of the OUL paradigm for studying memory updating impairments in AD-related pathology and identify flexible spatial updating under interference as a sensitive behavioral domain affected by amyloidosis.

## Introduction

The ability to update existing memories is essential to adapt to a changing environment. In everyday life, this process rarely involves learning entirely new information in isolation; instead, it often requires modifying previously acquired representations in light of new experience. Even though in aging brains most memories are not entirely novel, but rather modifications of pre-existing ones (Kredlow et al., 2018), it remains unclear how deficits in memory updating might contribute to the cognitive decline observed in Alzheimer’s disease (AD). A critical aspect of memory updating is the resolution of interference between competing and non-competing memory associations (Wahlheim and Zacks, 2024), a process that may be particularly relevant for spatial disorientation in AD but remains insufficiently explored.

In the clinical setting, cognitive impairment in AD is commonly assessed using tasks that probe episodic memory, executive function, and spatial orientation (Coughlan et al., 2018; Frisoni et al., 2025; Segen et al., 2025). These measures are informative for diagnosis and staging, but they do not directly isolate the neural mechanisms required to update previously stored information under conditions of interference (Wahlheim and Zacks, 2024). In parallel, many preclinical memory assays emphasize acquisition, recognition, or aversive learning, rather than flexible revision of previously acquired spatial representations. Preclinical models therefore offer an opportunity to examine memory updating under controlled conditions and relate this process to defined pathological features. Amyloidosis is currently among the earliest pathological markers, followed by tauopathy, synaptic loss, and neurodegeneration (Milà-Alomà et al., 2020; Hampel et al., 2021). The APP/PS1 transgenic mouse line, which expresses mutant human transgenes for APP (Swedish mutations) (Mullan et al., 1992) and PSEN1 (L166P mutation) (Moehlmann et al., 2002), develops cortical amyloid *β* (Aβ) deposition by 6 weeks and hippocampal deposition by 3–4 months (Radde et al., 2006). Cognitive impairments emerge by 7–8 months (Serneels et al., 2009), including deficits in spatial learning and reversal learning (Radde et al., 2006). However, behavioral impairment is not always linearly predicted by plaque burden, suggesting that amyloidosis may affect memory through broader synaptic and circuit-level consequences rather than plaque load alone (Cooper-Knock et al., 2012; Ovsepian et al., 2021; Davies et al., 2023; Hyman, 2023).

Updating stored memories requires the brain to resolve interference between previously learned and newly encountered information. This process is especially relevant for hippocampus-dependent memory, as previously stored representations must be reactivated, compared with new input, and either maintained or modified. Work in both humans and rodents has implicated hippocampal circuits in the handling of overlapping or competing memories, particularly when previously learned spatial information must be revised (Wichert et al., 2011; Josephs et al., 2017). In addition, the medial entorhinal cortex (mEC) is essential for spatial learning and memory through its grid cell network (Pilly and Grossberg, 2012; Malone et al., 2024), and it is among the earliest cortical regions to exhibit AD-related pathology (Silva and Martínez, 2023). Together, these observations suggest that tasks probing spatial memory updating may provide a useful experimental window into early hippocampal-entorhinal dysfunction in AD models.

Most memory tests for rodents focus on fear-based paradigms, which rely on strong emotional associations. In contrast, spatial memories, particularly those formed during free exploration, are more flexible and susceptible to modification (Bostock et al., 1991; Kentros et al., 2004). The objects in updated locations (OUL) paradigm allows assessment of how well spatial memories adapt when one familiar location is changed and later re-encountered in the context of competing old and updated spatial information (Kwapis et al., 2020). As a non-stressful and hippocampal-dependent task, it is well-suited for studying memory updating and interference resolution in AD models. Although there is no direct one-to-one human analog of this task, it captures processes that are relevant to broader clinical constructs, including spatial memory updating, interference resolution, and the flexible use of contextual information.

Sex differences in AD are well-established, with women accounting for nearly two-thirds of all AD cases and being almost twice as likely as men to develop the disease (Beam et al., 2018; Ferretti et al., 2018; Nebel et al., 2018). This disparity is only partially explained by differences in life expectancy. Biological factors such as sex hormones, genetics, and brain structure are thought to contribute to the increased vulnerability observed in women. Importantly, women with AD often exhibit higher amyloid and tau pathology and experience faster cognitive decline, particularly in episodic memory, compared to men (Laws et al., 2016). These findings highlight that sex should be factored into clinical and preclinical AD research. For this reason, evaluating males and females separately may help identify sources of vulnerability that are obscured in pooled analyses and improve the interpretability of preclinical findings.

To complement behavioral measures, immediate-early gene mapping can provide insight into the neuronal populations recruited during memory-related experiences (Minatohara et al., 2016; Gallo et al., 2018; Khalaf and Gräff, 2019). In particular, cFos expression is widely used as a marker of recent neuronal recruitment associated with novelty exposure, learning, and memory-related processing, including within hippocampal and entorhinal circuits (Rosen et al., 1998; Katche et al., 2009). In AD mouse models, altered cFos-defined ensemble recruitment has been linked to impaired memory retrieval (Christensen et al., 2013; Roy et al., 2016; Poll et al., 2020). Although cFos expression does not provide a direct measure of synaptic plasticity or real-time circuit dynamics, it can be used to identify regional patterns of neuronal recruitment that may relate to subsequent memory performance. In the context of spatial memory updating, such mapping could help link behavioral deficits to altered recruitment of hippocampal-entorhinal networks in AD models.

In the present study, we used the OUL paradigm to test whether APP/PS1 mice show altered spatial memory performance when previously learned and updated spatial representations compete. We further examined whether these effects varied with age and sex, and whether behavioral performance was associated with regional amyloid pathology and cFos expression following novelty exposure as a proxy of recent neuronal recruitment. Overall, APP/PS1 mice showed less reliable performance and weaker handling of competing spatial information than controls, suggesting that this paradigm may capture interference-sensitive aspects of spatial memory relevant to preclinical AD.

## Methods

All key resources used in this manuscript are listed in Supplementary Table 1.

### Mice

The subjects were adult female and male APP/PS1 mice (mutations: APP K670 M671delinsNL (Swedish) and PSEN1 L166P, genetic background: C57BL/6 J mice) obtained from Charité FEM, aged 4–11 months (see Supplementary Table 2 for details). The mice were housed together with their wild-type littermates in groups of 2–10 throughout their lives. They were kept in a ventilated scantainer in 12 h light/dark cycle with ad libitum access to food and water and controlled environmental conditions. All procedures were approved by the local authorities and ethics committee (LaGeSo Berlin, license numbers G0047/21), and in compliance with Charité FEM’s animal protection policy.

### Behavior procedure

The protocol was based on the objects in the updated location (OUL) paradigm, initially described by Wright et al. (2020), with some adjustments for our animal model. The experiments were conducted during light cycle. Handling and habituation were performed for 5 days. The OUL experiments were done on days 6 and 7. For this, we used a 50 cm × 50 cm open field arena. This consisted of three 15-min training sessions, a 5-min object displacement session, and a 10-min updating performance session, all 3 h apart. The first two training sessions occurred on day 6, and the rest on day 7. Object placements during training and object displacement sessions varied across different cohorts of mice to avoid potential pattern preference. Between behavior sessions, mice were housed together. On day 8 the mice were exposed to a novel open-field arena within the same experimental room. The floor and the walls were changed. There were no objects in the arena. To ensure a precise 90-min interval between environmental exploration and perfusion for optimal c-Fos quantification, exploration duration was set to 20 min. Due to logistical constraints of performing sequential perfusions within a single cohort, this duration was occasionally extended to a maximum of 25 min. This approach was prioritized over splitting cohorts across multiple days to minimize inter-day experimental variability.

### Object exploration quantification

Object memory was quantified based on the time mice spent exploring each object. For the object displacement session, the first 10 s of cumulative exploration (the sum of all interaction bouts) were used to calculate the discrimination index (DI). For the updating performance session, this window was extended to the first 20 s of cumulative exploration to account for the increased number of objects and ensure sufficient interaction with each stimulus. Using a fixed cumulative duration ensures uniformity in attention and engagement levels across genotypes. Exploration time was measured using a Python script (GitHub repository: https://github.com/caniko/oul-analysis) that analyzed body-part coordinates (nose, ears, tail base) extracted from a trained DeepLabCut network (Mathis et al., 2018). Network performance was evaluated on a held-out test set (Supplementary Table 3). After applying a likelihood threshold of *p* > 0.6, the test RMSE was 8.93 px, corresponding to approximately 6.43 mm. Low-confidence predictions (*p* ≤ 0.6) were interpolated from surrounding frames and excluded from network evaluation. The accuracy of the resulting automated exploration scoring was further validated against independent manual scoring (see below). Exploration was defined by two simultaneous criteria: the mouse’s nose coordinate had to be within a 5 cm radius from the circular border of the object, and its gaze had to be oriented toward the stimulus (within 120° from the nose), a threshold selected to avoid overly restrictive classification and to include whisker-mediated investigative behaviors. Specifically, the gaze vector was mathematically defined as the line segment originating from the midpoint between the ears and extending through the nose coordinates. Due to the tapered geometry of the objects and camera angle, this 5 cm object-to-nose measurement corresponds to a physical distance of approximately 3–4 cm from the object’s edge. This threshold was empirically validated through visual inspection (Supplementary Figure 1) to ensure that only active investigative behaviors, such as olfactory and tactile exploration, were recorded, while incidental “pass-through” events were excluded. To validate the automated scoring approach, a subset of 5 mice (balanced across genotype, sex, and age) was independently scored by a human observer blind to genotype and automated results. Manual scoring was performed on the object displacement and updating performance sessions. Because total exploration time per session was fixed by design, object exploration times were normalized within each session and expressed as proportions of total exploration time. Agreement between automated and manual scoring was therefore assessed at the level of individual object observations by comparing these normalized exploration proportions using Spearman correlation (*p* < 0.0001, Supplementary Figure 2), demonstrating a strong association between methods. In addition, Bland–Altman analysis revealed minimal systematic bias between methods (bias = −0.0003), with limits of agreement ranging from −0.138 to 0.137, indicating good agreement in the allocation of exploration across objects. To account for repeated measurements within mice, agreement was further evaluated at the level of individual mouse sessions by calculating the mean absolute difference in object-wise exploration proportions. The average error was low (mean ± SD = 0.028 ± 0.015), confirming consistent agreement between automated and manual scoring across animals. To ensure accuracy, objects were manually labeled, including their radius (as they were circular), and the meter-to-pixel ratio was determined using the makesense.ai tool (GitHub repository: https://github.com/SkalskiP/make-sense). The discrimination index was calculated as follows:


$$\mathrm{DI}=\frac{\left(\text{time exploring novel}-\text{time exploring familiar}\right)}{\left(\text{time exploring novel}+\text{time exploring familiar}\right)}\times 100\%$$

In cases where a mouse did not explore either of the two compared objects, the denominator equaled zero, and the DI could not be calculated. Assigning a DI of zero in these cases would incorrectly imply equal exploration of both objects, when in fact no exploration occurred. These instances were therefore excluded from the entire session, accounting for the variation in sample sizes across panels within the same figure.

### Immunohistochemistry

Each animal was euthanized using a mixture of xylazine (20 mg/kg) and ketamine (160 mg/kg), followed by transcardial perfusion 1.5 h after novel environment exposure with 1x PBS solution (10x PBS, ThermoFisher #70011044), and then with 4% paraformaldehyde (PFA; ThermoFisher #J19943. K2). The brains were extracted and stored in 4% PFA overnight at 4 °C for further fixation. The PFA was then replaced with 1x PBS until sectioning using a vibratome. For sectioning, the brains were split into left and right hemispheres, with the left hemisphere sectioned sagittally and the right hemisphere sectioned coronally into 40 μm-thick slices. The slices were then stored in 0.02% azide solution until immunostaining was performed.

To perform immunohistological quantifications of Aβ plaques, the Aβ 6E10 purified unconjugated antibody was used (1:500, #BioLegend 803,001). Two slices from each animal were used: one coronal slice containing the dorsal hippocampus (approximately −1.8 mm from Bregma) and one sagittal slice depicting the medial entorhinal cortex (mEC) and subiculum (Sub; approximately 3 mm laterally). Additionally, slices from different age and sex groups were selected for cFos (1:500, Cell Signaling #2250S) overlap quantification with NeuN (1:2000, Sigma-Aldrich #ABN91) and GFAP (1:500, Sysy #173004).

Slices were washed in 1x PBS, then incubated in 70% formic acid for 20 min for antigen retrieval, followed by three additional washes with 1x PBS. To block nonspecific binding, the slices were treated with a solution containing 3% bovine serum albumin (BSA), 8% normal goat serum (NGS) and 0.5% Triton-X in 1x PBS for 1 h at room temperature (RT). Slices were then incubated overnight at 4 °C with the primary antibody diluted in 1x PBS containing 5% NGS and 0.5% Triton-X. After overnight incubation and three 15-min washes with 1x PBS containing 0.5% Triton-X, the slices were incubated for 2 h at RT with the secondary antibody solution diluted 1:1000 in 1x PBS containing 4% NGS and 0.5% Triton-X. The sections were washed three times for 15 min in 1x PBS containing 0.5% Triton-X before being stained with DAPI (ThermoFisher 62,247) at 1:10,000 in 1x PBS for 20 min. Following DAPI staining, slices were washed three times for 10 min with 1x PBS, and mounted with Prolong Gold Antifade (ThermoFisher P36930) mounting media. Overview images were acquired with a custom-built Thorlabs EpiCerna fluorescence microscope with a 4x objective. Overlap quantification was performed on confocal image z-stacks acquired using a Leica SP5 confocal microscope (20x objective).

### Statistical analysis

For non-parametric comparisons between two groups, Mann–Whitney (MW) U test was used. For comparisons across three or more groups, Kruskal-Wallis test (KW) with Dunn’s post-hoc test was applied for comparisons in Aβ plaque pathology. The one-sample Wilcoxon test was used to assess whether the DI differed from zero. Holm-Bonferroni (HB) correction was applied separately within each genotype across the three memory type comparisons, as each genotype was treated as an independent family of comparisons. Within each genotype, the Wilcoxon matched-pairs signed rank test (or simply Wilcoxon test) was used to compare discrimination indices between object locations #3 and #1 across the sessions and to assess within-animal changes in spatial discrimination following the object relocation. Simple linear regression and Spearman’s rank correlation were used to evaluate correlation strength, while Binomial test was performed for associations in memory criteria performance between control and APP/PS1 groups. Holm-Bonferroni for multiple comparison correction was performed, with correction performed separately within each stratification (sex-based and age-based analyses treated as independent families, as these represent non-overlapping subgroups addressed in separate analyses). For the updating performance session with four objects, discrimination indices were analyzed using a two-way repeated measures ANOVA with Tukey’s post-hoc correction, to account for within-subject correlation across indices obtained from the same animal within the same session; where sphericity was violated, Greenhouse–Geisser correction was applied. Statistical significance was set at *p* < 0.05 and trend at *p* < 0.1.

## Results

### Amyloidosis impairs memory retention under spatial competition

**Figure 1:**
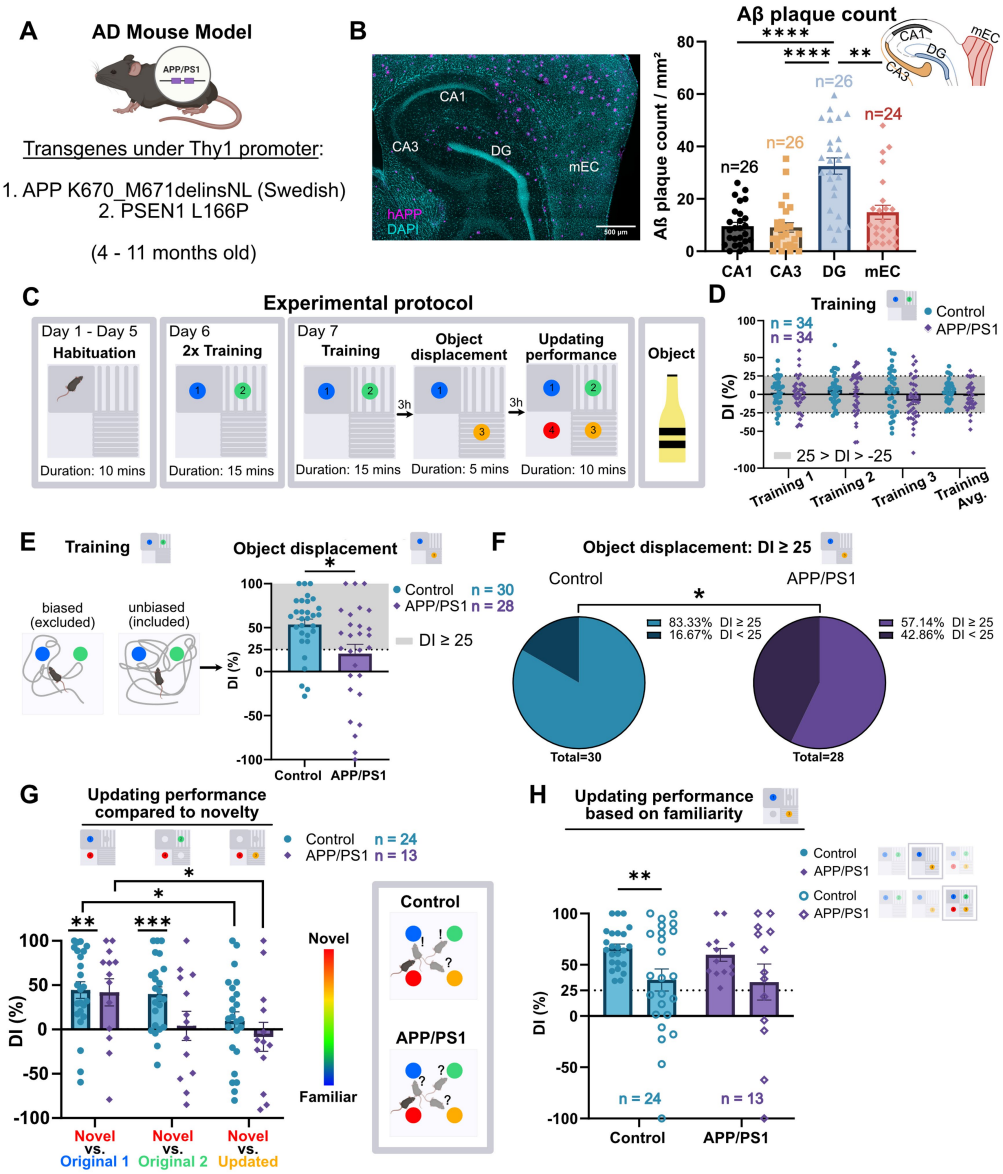
APP/PS1 mice show impaired ability to retain the original memory when faced with competing spatial information. **(A)** AD mouse model with double point mutation (APP/PS1). **(B)** Left: example of right HC stained for hAPP and with DAPI. Right: Aβ plaque count per mm2 for CA1, CA3 and DG (n = one slice per mouse; CA1 vs. DG: *p* < 0.0001, CA3 vs. DG: *p* < 0.0001, mEC vs. DG: *p* = 0.004, Kruskal-Wallis test with Dunn’s post-hoc test). **(C)** Schematic of experimental protocol consisting of 5 days of habituation to the room and OF. On day 6 and 7 behavioral experiments are conducted with identical objects (right) in different locations. **(D)** DI for the objects during all 3x training sessions and the average DI across all the training sessions. The shaded area indicates DI values of mice considered unbiased towards any of the 2x objects (original 1 and 2). **(E)** DI from object displacement session where one of the objects was in a new (updated) location. Here, only mice that were considered unbiased were included. Control mice demonstrated a DI significantly different from 0 (*n* = 30, *p* < 0.0001, One sample Wilcoxon test), APP/PS1 mice did not (*n* = 28, *p* = 0.067) with a significant difference between the groups (*p* = 0.015, MW test). **(F)** Pie chart showing the proportion of mice in each group that showed either DI ≥ 25 or DI < 25 for the object in the updated location (yellow) vs. original #1 (blue; *p* = 0.0010, Binomial test). **(G)** DI for three different object locations compared to the novel location. The colorbar indicates familiarity to novelty range of the objects (Novel vs. Original #1: p_control_ = 0.0004, p_APP/PS1_ = 0.129, Novel vs. Original #2, p_control_ = 0.0003, p_APP/PS1_ > 0.9999, Novel vs. Updated: p_control_ = 0.303, p_APP/PS1_ > 0.9999, one sample Wilcoxon test with HB post-hoc correction) with memory type differences (control: novel-O1 vs. novel-updated, *p* = 0.022; APP/PS1: novel-O1 vs. novel-updated, *p* = 0.032, ANOVA with Tukey’s post-hoc test), but none within genotype (*p* = 0.143) **(H)** Bar plot depicting DI between a familiar object and an updated spatial location across two sessions.Data are shown for both control and APP/PS1 mice during the initial object displacement and the subsequent re-exposure (p_control_ = 0.004; p_APP/PS1_ = 0.340, Wilcoxon test). Data are presented as mean ± SEM. * *p* < 0.05, ** *p* < 0.01, *** *p* < 0.001. See Supplementary Tables 4–6 for more statistical details. AD, Alzheimer’s disease; HC, hippocampus; hAPP, human amyloid precursor protein; Aβ, Amyloid β; CA, cornu ammonis; DG, dentate gyrus; mEC, medial entorhinal cortex; OF, open field; DI, discrimination index. Mouse schematic was created with BioRender.com.

To investigate how Aβ pathology impacts memory performance, we first established the regional distribution of Aβ deposition in APP/PS1 mice. Rather than serving as a simple marker of disease stage, plaque burden was evaluated as the context in which circuit- and behavior-level changes emerge. Aβ plaques were detectable in the hippocampus as early as 2 months of age (Supplementary Figure 3; Supplementary Table 2). We found Aβ plaque density to be significantly higher in dentate gyrus (DG) compared to CA1 (*p* < 0.0001, Figures 1A,B), CA3 (*p* < 0.0001), and medial entorhinal cortex (*p* = 0.004, Kruskal-Wallis with Dunn’s post-hoc correction).

To assess spatial memory function, mice were tested in an object-location memory task. To quantify animal behavior, we implemented improved animal tracking and automated scoring based on defined heuristics, enabling faster, unbiased, and reliable analysis of object-interaction behavior. During the initial training phase (Training, Figure 1C), we ensured that no intrinsic bias existed toward any of the object locations, by excluding from the study mice that showed, on average, a bias toward either of the two objects to which they were equally exposed (Figure 1D, see Supplementary Table 2 for animal numbers). When one of the familiar objects was moved (Figure 1C, Object Displacement), APP/PS1 mice (*n* = 28) showed significantly lower discrimination index (DI) compared to control littermates (*n* = 30, *p* = 0.015, Mann–Whitney U test [MW test thereafter], Figure 1E). Additionally, they showed no significant deviation from 0 in DI for displaced object location vs. original #1 (*p* = 0.067) while their control littermates did (*p* < 0.0001, one sample Wilcoxon test, Figure 1E). We also found that a significantly smaller proportion of APP/PS1 mice were able to discriminate compared to control littermates (*p* = 0.001, Binomial test; Figure 1F), with 43% of APP/PS1 mice not being able to discriminate (DI < 25%) compared to only 17% of control mice. A discrimination threshold of DI ≥ 25% was adopted based on prior OUL studies (Kwapis et al., 2020; Wright et al., 2020) in which young control animals reliably performed at or above this level. Mice with DI < 25% were therefore excluded from subsequent updating analyses. Although the original studies did not formally derive this cutoff, it reflects an empirically observed level of performance associated with successful memory updating. Because updating depends on successful encoding and retention of the initial displacement, failure to detect that displacement makes subsequent updating measures difficult to interpret. The results without the previously mentioned exclusion have also been interpreted and presented in the Supplementary Figures 4–6. In the subsequent testing session (Figure 1C, Updating performance), mice explored an arena containing three familiar object locations (two at the original and one at the updated location) and a fourth object at a novel location. Control mice successfully discriminated between the novel and both original locations (Novel vs. Original #1: *p* = 0.0004, Novel vs. Original #2, *p* = 0.0003), but not the updated location (*p* = 0.303, one sample Wilcoxon test, Figure 1G). However, APP/PS1 mice showed no significant discrimination between the novel location and any of the familiar locations (Novel vs. Original #1: *p* = 0.129, Novel vs. Original #2: *p* > 0.999, Novel vs. Updated: *p* > 0.999). Further analysis revealed that only control mice exhibited a significant DI decrease when comparing updated object locations to the original #1 between the two sessions of exposure to those two objects (control: *p* = 0.004; APP/PS1: *p* = 0.340, Wilcoxon test, Figure 1H), consistent with integration of updated spatial information in control but not APP/PS1 mice. Importantly, we did not observe any differences in total displacement or median speed between APP/PS1 mice and their control littermates (Supplementary Figure 7).

### Exploration latency and its relationship to memory performance

To investigate potential behavioral factors influencing memory performance, we analyzed exploration latency defined as the time required to reach the total object exploration criteria. Different exploration time criteria were set for different sessions: in the object displacement session, exploration was assessed over the first 10 s, while in the updating performance session, it was extended to 20 s to account for the increased number of objects and ensure adequate exploration (Vogel-Ciernia and Wood, 2014; Traschütz et al., 2018). Using consistent exploration time ensures uniformity in the level of attention and engagement given to each object, reducing variability in the exploration process and allowing for more accurate comparisons across objects.

**Figure 2:**
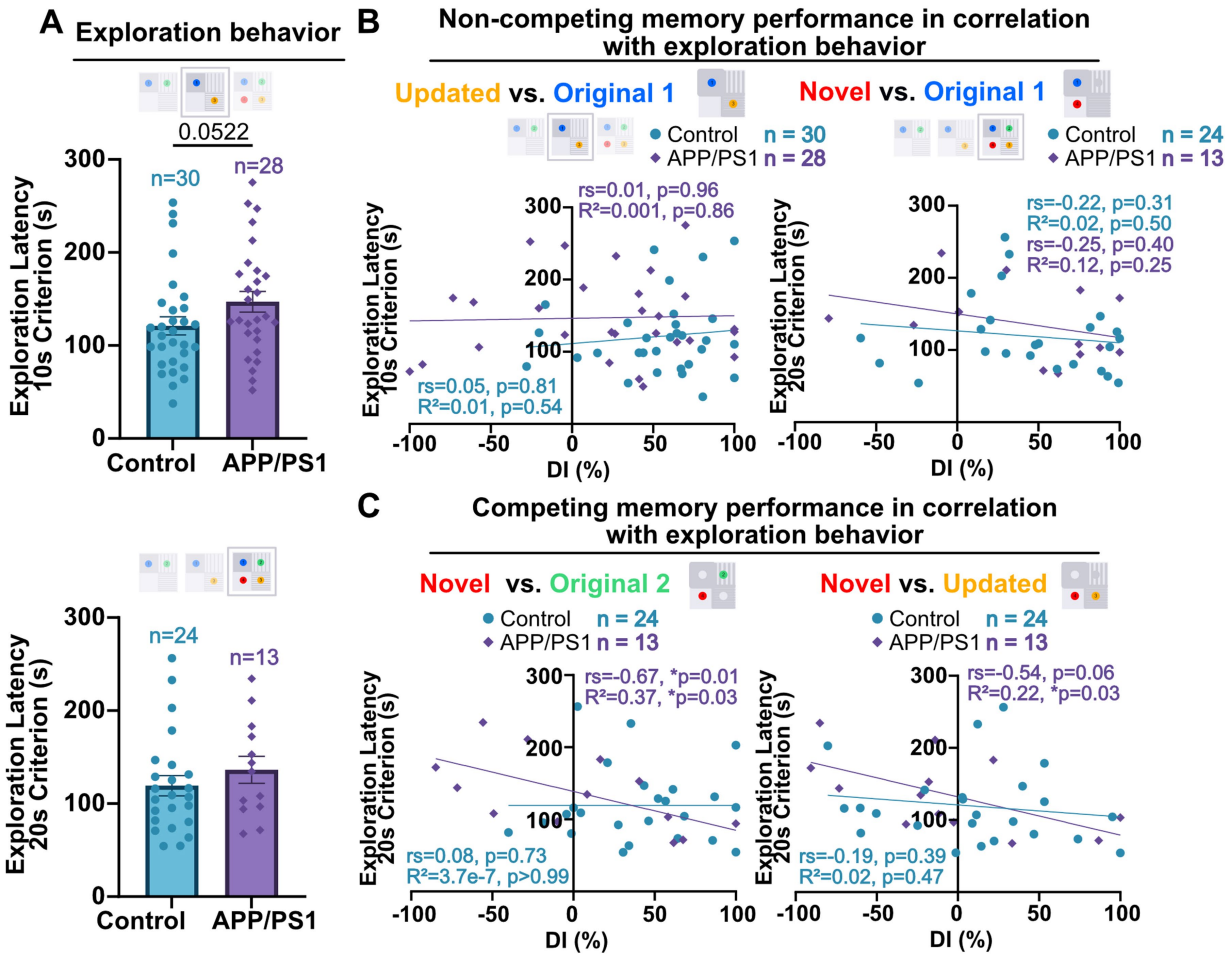
Exploration latency in APP/PS1 mice is selectively associated with weaker memory performance involving competing spatial elements. **(A)** Exploration latency is shown as time taken to reach the object exploration criteria during object displacement (upper panel: *p* = 0.052, MW test) and updating performance session (lower panel: *p* = 0.306, MW test). (B, C) Scatterplot showing the relationship between exploration latency and discrimination index (DI) between objects with **(B)** non-competing spatial elements and **(C)** competing spatial elements. The solid line represents the simple linear regression fit. Spearman’s rank correlation coefficient (r_s_) and coefficient of determination from linear regression (*R*^2^) statistics, along with their respective *p*-values and number of mice (*n*), are indicated directly on the graphs. Individual data points represent single mice. Data are presented as mean ± SEM. * *p* < 0.05, ** *p* < 0.01. See Supplementary Table 7 for more statistical details.

APP/PS1 mice showed no significant difference in exploration latency compared to control littermates during either the object displacement session (*p* = 0.052, MW test, Figure 2A top), or the updating performance session (*p* = 0.307, MW test; Figure 2A bottom). To further explore the relationship between exploratory behavior and memory performance, we analyzed the correlation between exploration latency and memory scores for both control and APP/PS1 genotypes. There was no significant correlation between the exploration latency and memory performance involving non-competing spatial elements in either APP/PS1 mice (Updated vs. Original #1: *p* = 0.963, Figure 2B left; Novel vs. Original #1: *p* = 0.401, Figure 2B right) or control mice (Updated vs. Original #1: *p* = 0.814; Novel vs. Original #1: *p* = 0.310, Spearman correlation). In contrast, exploration latency was significantly associated with competing memory performance in APP/PS1 mice. For the Novel vs. Original #2 comparison, both Spearman correlation (*p* = 0.015) and linear regression (*p* = 0.028) reached significance (Figure 2C left). For Novel vs. Updated comparison, the linear regression was significant (*p* = 0.033) while Spearman correlation showed a trend (*p* = 0.061; Figure 2C right). In control mice, no association was found between exploration latency and either competing comparison (Novel vs. Original #2: *p* = 0.725; Novel vs. Updated: *p* = 0.386; Spearman correlation). This pattern suggests that exploration latency is selectively related to memory performance when spatial information involves competing elements.

### Sex differences in spatial discrimination, memory updating, and amyloidosis

**Figure 3:**
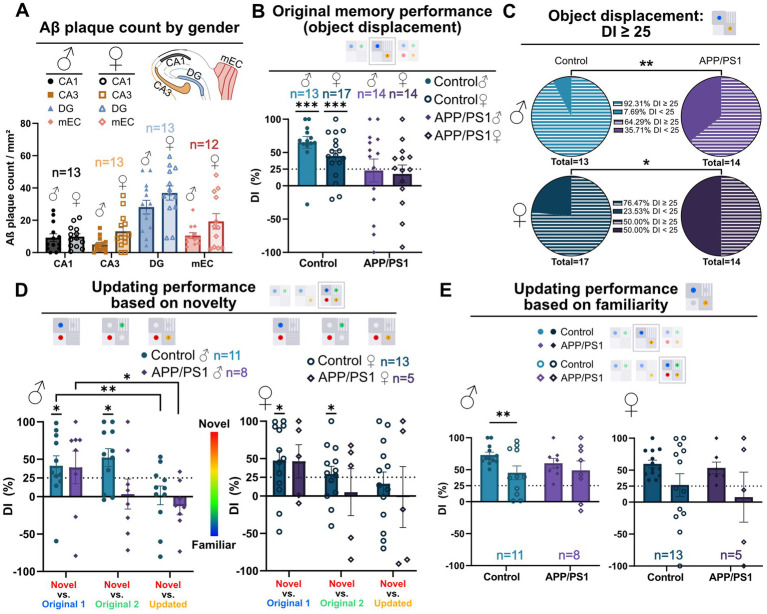
Male and female APP/PS1 mice exhibit comparable spatial memory impairments. **(A)** Amyloid plaque burden differed significantly across hippocampal subregions (*p* < 0.0001) and between sexes (*p* = 0.005, two-way ANOVA), with no significant interaction. However, no significant pairwise differences between males and females were identified in any individual region after Bonferroni correction (all *p* > 0.221). **(B)** Discrimination between objects in original location #1 and updated location (#3) between the genotypes and sexes (Males: *p*control = 0.0006, *p*APP/PS1 = 0.345; Females: *p*control = 0.0006, *p*APP/PS1 = 0.345; one sample Wilcoxon test, Males: *p* = 0.147, Females: *p* = 0.147, MW test). **(C)** Pie chart showing the proportion of mice in each group that showed either DI ≥ 25 or DI < 25 for the object in updated location (yellow) vs. original #1 (blue) for males and females (Binomial test, Males: *p* = 0.0030; Females: *p* = 0.0280). **(D)** Updating performance for male and female mice based on novelty. First panel (Males): Novel vs. Original #1: *p*control = 0.037, *p*APP/PS1 = 0.586; Novel vs. Original #2: *p*control = 0.020, *p*APP/PS1 > 0.999; Novel vs. Updated: pcontrol = 0.577, pAPP/PS1 = 0.766, one sample Wilcoxon test; memory type: Novel vs. Original #1 and Novel vs. Updated: pcontrol = 0.008, pAPP/PS1 = 0.032, genotype: *p* = 0.167, ANOVA with Tukey’s post-hoc test. Second panel (Females): Novel vs. Original #1: pcontrol = 0.024, pAPP/PS1 = 0.563; Novel vs. Original #2: pcontrol = 0.027, pAPP/PS1 > 0.999; Novel vs. Updated: *p*control = 0.340, pAPP/PS1 > 0.999, one sample Wilcoxon test; memory type: *p* = 0.077, genotype: *p* = 0.529, ANOVA with Tukey’s post-hoc test. **(E)** Bar plot depicting DI between a familiar object and an updated spatial location across two sessions (updating performance based on familiarity) for both control and APP/PS1 male (left panel) and female (right panel) mice during the initial object displacement and the subsequent re-exposure (Males: pcontrol = 0.0098, pAPP/PS1 = 0.742, Females: *p* control = 0.293, pAPP/PS1 = 0.3125, Wilcoxon test). Individual data points represent single mice. Data are *p* resented as mean ± SEM with each dot rep resenting one mouse/slice * *p* < 0.05, ** *p* < 0.01, *** *p* < 0.001. See Supplementary Tables 4, 6, 8 for more statistical details. HC, Hippocampus; hAPP, human amyloid precursor protein; Aβ, Amyloid β; CA, cornu ammonis; DG, dentate gyrus; mEC, medial entorhinal cortex; OF, open field; DI, discrimination index; ROI, region of interest.

To explore potential sex-dependent effects on memory performance, we first characterized amyloid plaque distribution across hippocampal subregions and medial entorhinal cortex in male and female APP/PS1 mice. We found a significant main effect of sex on amyloid pathology (*p* = 0.005, ANOVA, Figure 3A); however, no significant pairwise differences were identified after Bonferroni’s post-hoc correction (all *p* > 0.221). In the object displacement session, where one of the familiar objects (original #2) was displaced, neither male (*p* = 0.345) nor female APP/PS1 mice (*p* = 0.345) showed a DI significantly different from zero whereas both male (*p* = 0.0006) and female (*p* = 0.0006, one sample Wilcoxon test, Figure 3B) control mice did. However, there were no significant differences between APP/PS1 and control groups for either males (*p* = 0.15) or females (*p* = 0.15, MW test). Notably, a significantly greater proportion of APP/PS1 mice fell below the 25% discrimination threshold compared to control mice in both sexes: among females, 50.00% of APP/PS1 mice had DI < 25% compared to 23.53% of controls (*p* = 0.028, binomial test, Figure 3C), and among males, 35.71% of APP/PS1 mice had DI < 25% compared to 7.69% of controls (*p* = 0.003).

During the updating performance test, male mice showed a significant effect of memory type (*p* = 0.01, ANOVA, Figure 3D left), but no significant effect of genotype (*p* = 0.167). Tukey’s post-hoc test revealed that both control (*p* = 0.008) and APP/PS1 (*p* = 0.032) males showed a significant difference between Novel vs. Original 1 and Novel vs. Updated comparisons, suggesting that both genotypes retained some memory of the original location. One-sample Wilcoxon tests confirmed that control males showed a discrimination index significantly different from zero for Novel vs. Original 1 (*p* = 0.038) and Novel vs. Original #2 (*p* = 0.020), whereas APP/PS1 males did not (Novel vs. Original #1: *p* = 0.586, Novel vs. Original #2: *p* > 0.999). In female mice, no significant effects were found for memory type (*p* = 0.08), genotype (*p* = 0.529, ANOVA, Figure 3D right).

When analyzing memory updating based on familiarity (original #1 object location), only control male mice showed significant reduction in DI between the object displacement and updating performance sessions (*p* = 0.01, Figure 3E). No significant change in DI between sessions was observed in control female mice (*p* = 0.293) or in APP/PS1 male (*p* = 0.742) mice or female (*p* = 0.313, Wilcoxon test) mice.

### Age-dependent differences in memory performance and associated hippocampal pathology

**Figure 4:**
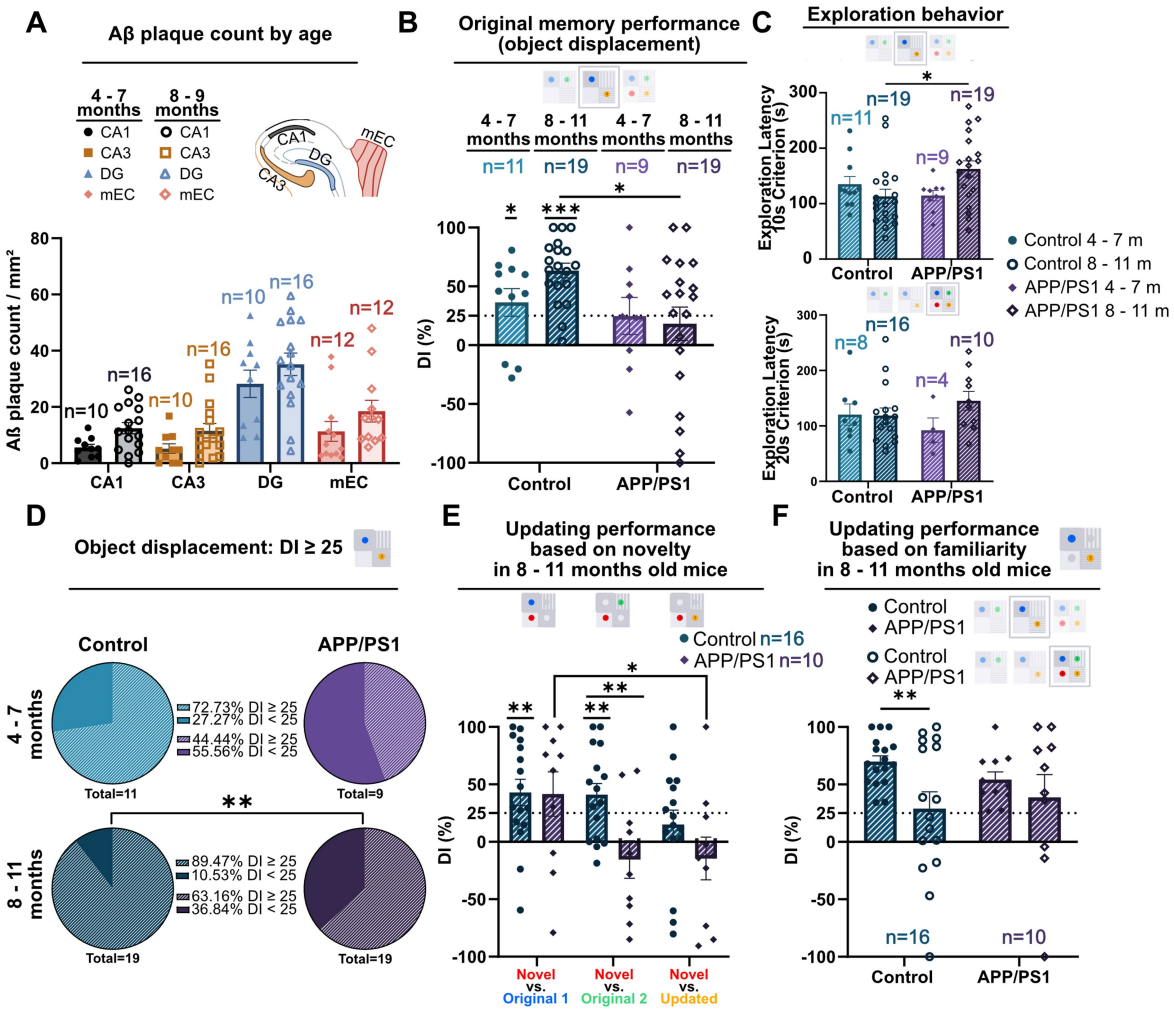
Age-dependent memory performance differences between control and APP/PS1 mice. **(A)** Amyloid plaque count in CA1, CA3, DG, and mEC in comparison with age, 4–7 vs. 8–9-months-old mice (*p* = 0.004, ANOVA; CA1: *p* = 0.579, CA3: *p* = 0.700, DG: *p* = 0.556; mEC: 0.518). **(B)** Discrimination between objects in original location # 1 and updated location between the different experimental age groups (4–7 and 8–11-months-old) during object displacement (4–7 months: *p*_control_ = 0.041, *p*_APP/PS1_ = 0.328; 8–11 months: *p*_control_ = 0.0004, *p*_APP/PS1_ = 0.328, one-sample Wilcoxon test; 4–7-months-old: *p* = 0.503; 8–11-months-old: *p* = 0.028, MW test). **(C)** Exploration behavior in different age groups quantified by object exploration latency during object displacement (upper panel: 4–7 months: *p* = 0.710, 8–11 months: *p* = 0.024, MW test), and updating performance session (bottom panel: 4–7 months: *p* = 0.461, 8–11 months: *p* = 0.365, MW test). **(D)** Pie chart showing the proportion of mice in each group that showed either DI ≥ 25 or DI < 25 for the object in updated location (yellow) vs. original #1 (blue) for 4–7 and 8–11-months-old mice (4–7 months: *p* = 0.069; 8–11 months: *p* = 0.002, Binomial test). **(E)** Updating performance for 8–11-months-old control and APP/PS1 mice (Novel vs. Original #1: pcontrol = 0.005, pAPP/PS1 = 0.246; Novel vs. Original #2: pcontrol = 0.005, pAPP/PS1 = 0.863; Novel vs. Updated: pcontrol = 0.159, pAPP/PS1 = 0.863, one-sample Wilcoxon test; genotype effect: Novel vs. Original #2, *p* = 0.009, memory type in APP/PS1: Novel vs. original #1 and Novel vs. updated, *p* = 0.0449, ANOVA with Tukey’s post-hoc test). **(F)** Updating performance based on familiarity in 8–11-months-old mice (pcontrol = 0.005; pAPP/PS1 = 0.770, Wilcoxon test). Individual data points represent single mice. Data are presented as mean±SEM. * *p* < 0.05, ** *p* < 0.01, *** *p* < 0.001. See Supplementary Tables 4, 6, 9 for more statistical details. HC, hippocampus; hAPP, human amyloid p recursor protein; CA, cornu ammonis; DG, dentate gyrus; mEC, medial entorhinal cortex; OF, open field; DI, discrimination index; ROI, region of interest.

Advanced age is the most significant risk factor for Alzheimer’s disease, with numerous studies demonstrating a strong correlation between increasing age and the incidence and prevalence of the disorder (Xia et al., 2018). We examined memory performance in the OUL, comparing young adults (4–7-months-old) to early middle-aged adults (8–11-months-old), referred to hereafter as the younger and older groups, respectively. To assess whether these behavioral differences were correlated with Aβ pathology, we quantified Aβ plaque density across hippocampal subregions and the entorhinal cortex in both age groups. Two-way ANOVA revealed a significant main effect of age of plaque burden on hippocampal and mEC regions (*p* = 0.004, Figure 4A). However, no significant pairwise differences were identified after Tukey’s post-hoc test. During the object displacement phase, both younger and older control mice displayed DIs significantly different from zero (4–7 months: *p* = 0.041, 8–11 months: *p* = 0.0004, one sample Wilcoxon test, Figure 4B), indicating successful discrimination of the displaced object. In contrast, neither younger nor older APP/PS1 mice exhibited discrimination significantly different from zero (4–7 months: *p* = 0.328, 8–11 months: *p* = 0.328). A significant difference between genotypes was observed in the older age group (*p* = 0.028, MW test), but not in the younger cohort (*p* = 0.503).

We next examined exploration behavior as a potential indicator of memory performance. Older APP/PS1 mice exhibited increased latency to explore objects during object displacement compared to their age-matched controls (*p* = 0.024, MW test, Figure 4C top). In contrast, no significant latency difference was observed during the updating performance test (4–7 months: *p* = 0.461, 8–11 months: *p* = 0.365, MW test, Figure 4C bottom). To further parse these age effects, we evaluated individual performance using a criteria-based approach as before (DI ≥ 25). A significantly lower proportion of older APP/PS1 mice met this threshold compared to age-matched controls (8–11 months: *p* = 0.002, control DI ≥ 25: 89.47%, APP/PS1 DI ≥ 25: 63.16%, binomial test, Figure 4D), suggesting that while some APP/PS1 mice retained the ability to discriminate displaced object location, a significantly greater proportion failed to do so compared to age-matched controls. In contrast, although only 44.44% of younger APP/PS1 mice met the criterion, the difference from controls did not reach statistical significance (4–7 months: *p* = 0.069, control DI ≥ 25: 72.73%, APP/PS1 DI ≥ 25: 44.44%, binomial test, Figure 4D top,). Subsequently, we focused specifically on the memory updating performance session in the older cohort. During the updating performance test in older mice, a significant main effect of genotype was observed (*p* = 0.009, ANOVA; Novel vs. Original #2: *p* = 0.009, Tukey’s post-hoc test, Figure 4E), in addition to an effect of memory type (*p* = 0.006, ANOVA; APP/PS1: Novel-Original #1 vs. Novel-Updated, *p* = 0.0449, Tukey’s post-hoc test). While older control mice showed a DI significantly different from zero for comparisons between the novel location and both original object locations (Novel vs. Original #1: *p* = 0.005, Novel vs. Original #2: *p* = 0.005, one sample Wilcoxon test, Figure 4E), older APP/PS1 mice did not reach significance for any comparison (Novel vs. Original #1: *p* = 0.246; Novel vs. Original #2: *p* = 0.863; Novel vs. Updated: *p* = 0.863). Similar results were obtained when analyzing memory performance in older mice based on familiarity. In controls, the DI between Original #1 and updated object locations significantly decreased upon re-exposure (*p* = 0.005, Wilcoxon test, Figure 4F), whereas APP/PS1 mice did not (*p* = 0.770), consistent with reduced flexibility in updating spatial memory based on familiarity.

### Correlations between c-Fos expression, amyloid pathology, and spatial memory performance

**Figure 5:**
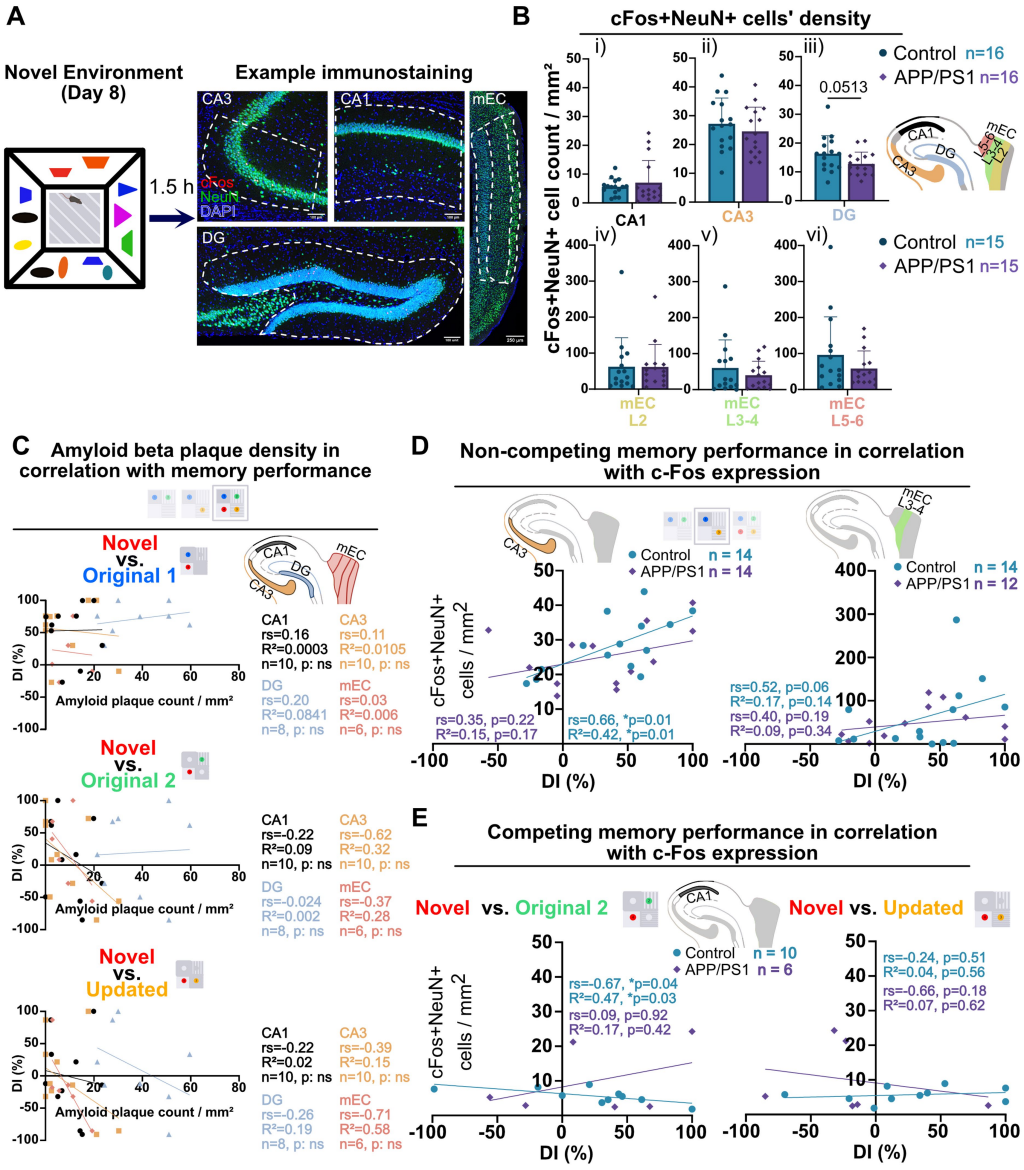
Positive correlation present between non-competing memory and c-Fos expression in CA3 in control mice. **(A)** Schematic of a novel environment used (left) and example of immunofluorescence imaging of HC (CA1, CA3, and DG) and mEC showing cFos, NeuN, and DAPI markers (right). **(B)** cFos+NeuN+ cells’ density in HC (i, CA1; ii, CA3; and iii, DG) and mEC layers (iv, 2; v, 3–4; and vi, 5–6) compared between control and APP/PS1 mice (CA1 *p* = 0.305, CA3 *p* = 0.381, DG *p* = 0.051, mEC L2 *p* = 0.539, mEC L3-4 *p* = 0.775, mEC L5-6 *p* = 0.436, MW test). **(C–E)** Scatter plots with simple linear regression and correlation between **(C)** Aβ plaque density in HC (CA1, CA3 and DG) and mEC and the updating memory performance, **(D)** c-Fos expression and memory with non-competing spatial elements, and **(E)** c-Fos expression and memory with competing spatial elements in control (blue) and APP/PS1 (purple) mice. Spearman’s rank correlation (r_s_) and coefficient of determination from linear regression (*R*^2^) statistics, along with their respective *p*-values and number of mice (*n*), are indicated directly on the graphs. Individual data points represent single mice. Data are presented as mean± SEM. * *p* < 0.05, ** *p* < 0.01. See Supplementary Tables 4, 10 for more statistical details. HC, hippocampus; hAPP, human amyloid precursor protein; CA, cornu ammonis; DG, dentate gyrus; mEC, medial entorhinal cortex. Mouse schematic was created with BioRender.com.

Novel-environment exposure is commonly used to probe recent neuronal recruitment in hippocampal-entorhinal circuits, and altered immediate-early gene induction has been reported in AD mouse models. To connect behavioral impairments with neural regional patterns of neuronal recruitment, we examined c-Fos expression in APP/PS1 mice during novel spatial exploration, with the aim of identifying activity patterns associated with memory performance. Therefore, in a subset of mice (control: *n* = 16, APP/PS1: *n* = 16) we added a Day 8 to the OUL paradigm, during which mice were exposed to a novel environment with new proximal cues (Figure 5A). This was performed to assess neuronal activation following novelty exposure and its possible correlation to memory performance of the previous day. Immunostaining for cFos and NeuN revealed that most cFos-positive cells (cFos+) were also positive for NeuN (NeuN+), suggesting that spatial exploration predominantly recruits neuronal populations rather than glia (Supplementary Figures 8–10). Although no significant differences were found in cFos+ neuron density between genotypes in either the hippocampus or mEC (Figure 5B), there was a trend towards reduced cFos+ neuron density in DG (*p* = 0.051, MW test).

Since the behavioral phenotype in our APP/PS1 cohort showed age- and sex-related heterogeneity, we asked whether novelty-induced c-Fos recruitment might also vary across these factors. Therefore, we also evaluated sex- and age-dependent differences in c-Fos expression in our cohort of mice. We found a significant decrease in cFos+ neuron density in the DG of APP/PS1 female mice compared to their control littermates (Supplementary Figure 11A) and a significant increase in cFos+ neuron density in the mEC layer 2 in younger APP/PS1 mice compared to control littermates (Supplementary Figure 11B).

Based on the well-established roles of hippocampal and mEC subregions for spatial memory, we then investigated how the patterns of plaque deposition and novelty-related cFos expression relate to memory performance in the paradigm used in this study. We first examined the relationship between amyloid plaque area and memory performance in the OUL paradigm. We found no statistically significant correlation between amyloid deposits and memory performance for either non-competing memory (*p* > 0.486 for all subregions, Figure 5C, first panel) or competing memory (*p* > 0.233 for all subregions, Figure 5C second and third panel) across all analyzed areas. However, the CA3 region showed a trend toward a negative association with one competing comparison (Novel vs. Original #2: *p* > 0.06, for both Spearman correlation and linear regression), whereas mEC showed a trend in correlation only (*p* = 0.078; *p* = 0.136, linear regression). Finally, we examined how c-Fos expression in hippocampal and mEC subregions following novel environment exposure correlated with memory performance in the OUL task. In control mice, increased CA3 c-Fos expression was significantly correlated with better memory performance (*p* = 0.012, Spearman correlation; *p* = 0.012, linear regression, Figure 5D left), whereas c-Fos-expression in mEC layers 3–4 was not (*p* = 0.061; *p* = 0.142, linear regression, Figure 5D right). In APP/PS1 mice, however, neither region showed significant correlation to memory performance during the object displacement session (CA3: *p* = 0.216, mEC: *p* = 0.194, Spearman correlation; CA3: 0.176, mEC: *p* = 0.345, linear regression). In CA1, c-Fos expression showed a significant correlation with updating memory performance involving one of the competing elements in control mice (Novel vs. Original #2: *p* = 0.039; *p* = 0.029, linear regression; Novel vs. Updated: *p* > 0.506 for both Spearman correlation and linear regression), unlike in APP/PS1 mice (Novel vs. Original #2: *p* > 0.424, Figure 5E left; Novel vs. Updated: *p* > 0.175 for both Spearman correlation and linear regression, Figure 5E right) in which no correlation was observed with either competing memory comparison.

## Discussion

This study provides novel insights into how competitive and non-competitive memory associations are disrupted in AD, an underexplored aspect of AD-related cognitive dysfunction. Using the Objects in Updated Locations (OUL) paradigm, we found that APP/PS1 mice showed weaker and less reliable spatial memory performance than control littermates, particularly when previously learned and updated spatial representations had to be handled in competition. Rather than indicating a uniform deficit across all measures, the data point to reduced reliability of performance and weaker handling of interference in the APP/PS1 group. The study further identifies age- and sex-related heterogeneity across behavioral and c-Fos-based measures and shows that plaque burden alone did not explain the observed behavioral variation.

### Competing spatial memory performance in APP/PS1 mice

A major aim of this study was to determine whether amyloidosis alters memory performance when old and updated spatial representations must be weighed against one another The OUL paradigm is useful in this context because it distinguishes initial detection of spatial change from later performance when a previously displaced location competes with updated information, thereby probing memory updating under interference rather than novelty detection alone (Kwapis et al., 2020; Wright et al., 2020). In the initial object-displacement session, APP/PS1 mice showed weaker performance than controls, consistent with an early difficulty in detecting or stabilizing the displaced location. This is also consistent with the literature of AD preclinical models in novel object location tests (Hulshof et al., 2022; Zhang et al., 2023; Park et al., 2025) or spatial memory tasks (Radde et al., 2006; Serneels et al., 2009; Viana da Silva et al., 2019). Importantly, the criteria-based analysis further showed that a larger proportion of APP/PS1 mice failed to reach successful discrimination, indicating that the phenotype was expressed not only as a shift in mean performance but also as reduced reliability across individuals.

In the following session for updating performance measurement, we excluded the mice based on the rationale that spatial memory updating relies on the successful consolidation of foundational information. If a mouse fails to show memory for the initial displacement, subsequent updating results become uninterpretable. During the updating phase, control mice discriminated the novel location from the stable original location and showed a reduction in discrimination between the original and updated locations across sessions, consistent with flexible adjustment of spatial representations. APP/PS1 mice, by contrast, showed weaker performance in comparisons that involved competing spatial elements and did not show the same across-session adjustment. At the same time, the direct genotype differences in the four-object updating session were modest, and several comparisons did not reach significance. For this reason, we avoid interpreting the data as evidence for a complete failure of updating in APP/PS1 mice. Instead, the results support a more circumscribed interpretation: APP/PS1 mice appear less able to maintain reliable performance when previously learned and updated spatial information compete, especially once the task requires resolution of overlapping representations. This interpretation is broadly compatible with previous work implicating hippocampal circuitry in interference resolution and flexible use of stored spatial information. Prior studies have linked CA3- and CA1-dependent processes to pattern completion, discrimination of overlapping representations, and retrieval of competing episodic or spatial contexts (Kesner and Rolls, 2015; Dimsdale-Zucker et al., 2018; Gonzalez et al., 2019; Zou et al., 2023). Our study does not directly test those circuit mechanisms, but the behavioral pattern is consistent with the idea that amyloidosis affects hippocampus-dependent processing most clearly when the task requires flexible weighting of conflicting spatial traces rather than simple exposure to novelty alone. Indeed, resolving interference between overlapping spatial representations is thought to place substantial demands on hippocampal circuits even under healthy conditions (Kwapis et al., 2020; Wright et al., 2020; Wahlheim and Zacks, 2024) and the OUL paradigm was specifically designed to probe this capacity. That amyloidosis selectively compromises performance under these demanding conditions, while leaving simpler spatial discrimination relatively more intact, underscores the sensitivity of interference resolution as a behavioral readout of hippocampal dysfunction.

### Exploration latency and memory performance

We further examined exploration latency as a complementary behavioral measure. APP/PS1 mice did not show a significant overall increase in latency across all sessions, although older APP/PS1 mice did take longer than age-matched controls to reach the exploration criterion during object displacement. More importantly, exploration latency showed a significant negative association with discrimination performance in APP/PS1 mice under conditions involving competing spatial information, supported by both correlation and regression analyses for one comparison and by regression for the other in one of the two comparisons involving competing spatial information, though this relationship was only supported by a trend in correlation analysis. Discrepancies between linear regression and Spearman correlation outcomes likely reflect the sensitivity of regression to the magnitude of individual data points. No such associations were observed for non-competing comparisons in either APP/PS1 or in controls. The specificity of this potential association suggests that the behavioral relevance of latency may emerge most clearly when task demands are higher. At the same time, exploration latency should be interpreted cautiously. Increased latency may reflect reduced exploratory drive (Ratuski et al., 2021), altered attentional engagement (Cimadevilla et al., 2001) or cognitive processing during object interaction (Gallagher and Burwell, 1989), anxiety-related hesitation (Prut and Belzung, 2003), or the greater cognitive demands imposed by interference between competing spatial traces (Puthusseryppady et al., 2024). While these variables should be taken into account as limitations that may have impacted performance, the fact that this latency correlated specifically with poor scores in the transgenic mice (but not the controls) suggests it is more than a general exploratory deficit. Additionally, the absence of genotype differences in total displacement and median speed argues against a simple locomotor explanation, but it does not eliminate these other possibilities. We therefore view latency not as a direct readout of memory updating, but as a complementary behavioral measure that may become particularly informative when considered together with performance under interference.

Our interpretation is also compatible with earlier work in AD models linking longer or less efficient exploration to impaired cognitive flexibility, inefficient search strategies, or altered decision making (Kim et al., 2021; Miller et al., 2021; Daumas et al., 2023; Eraslan Boz et al., 2024). In addition, Poll et al. (2020) showed that novelty-like neuronal ensembles can be aberrantly recruited during memory recall in an AD mouse model, which may be relevant to understanding why exploration latency and memory performance showed condition-specific associations in the present study, though we did not directly assess ensemble-level activity.

### Sex- and age-related heterogeneity in vulnerability

Because sex and age are both relevant modifiers of Alzheimer-related phenotypes, we examined whether performance in the OUL paradigm varied across these factors. The sex-stratified analyses suggest heterogeneity rather than a single uniform pattern. In the displacement session, neither male nor female APP/PS1 mice showed discrimination indices significantly different from zero, whereas both male and female controls did. Criteria-based analyses (taking mice with DI > 25% in the initial session) further showed that a larger proportion of APP/PS1 mice fell below the discrimination threshold in both sexes. During the updating session, male mice showed a significant effect of memory type, whereas female mice did not. In males, both genotypes differentiated between the most familiar original location and the updated location, suggesting that male APP/PS1 mice still showed some relative differentiation between spatial comparisons. However, only control males showed discrimination indices significantly above zero for individual comparisons, whereas APP/PS1 males did not. This dissociation between relative differentiation within the task and absolute performance above chance is consistent with the interpretation of reduced reliability rather than the complete absence of spatial memory in APP/PS1 mice.

Sex differences in AD are well documented clinically and experimentally (Laws et al., 2016; Beam et al., 2018; Ferretti et al., 2018; Nebel et al., 2018), and some APP/PS1 studies have reported greater pathological or cognitive vulnerability in females (Kim et al., 2015; Jiao et al., 2016). In the present dataset, however, the sex effects were mixed: criteria-based analyses indicated that both male and female APP/PS1 mice showed a significantly higher proportion of unsuccessful performers compared to their same-sex controls, suggesting the deficit was not confined to one sex. When analyzing memory updating based on familiarity, only control males showed a significant reduction in DI between sessions, whereas control females and APP/PS1 mice of both sexes did not, adding a further dimension to the sex-dependent pattern. We therefore interpret the present results as evidence for sex-related heterogeneity in phenotype expression rather than for a clear female-specific impairment. These observations underscore the importance of reporting and analyzing sex as a biological variable in preclinical AD research, as behavioral phenotypes may manifest differently across sexes and could be obscured in pooled analyses.

The age-stratified analyses were somewhat clearer. Older APP/PS1 mice showed poorer object-displacement performance than age-matched controls and took longer to reach the exploration criterion, whereas younger APP/PS1 mice did not differ significantly from younger controls. Criteria-based analyses similarly indicated a larger fraction of unsuccessful performers in the older APP/PS1 group. During the updating phase, a significant main effect of genotype was observed in older mice, with a significant difference between genotypes in the Novel vs. Original #2 comparison. Older control mice retained discrimination for both original locations and showed across-session changes consistent with updating, whereas older APP/PS1 mice did not show significant discrimination in any comparison. Notably, the genotype difference emerged specifically for the comparison involving Original #2, the object whose location had been displaced during the earlier session and therefore carried overlapping spatial traces. This specificity is consistent with accumulating evidence that amyloidosis preferentially affects the handling of competing or overlapping spatial representations rather than spatial memory retrieval more broadly (Palmer and Good, 2011; Poll et al., 2020), and extends this view to a freely exploratory spatial memory updating paradigm. This direct genotype effect, which was not apparent in the pooled or sex-stratified analyses, suggests that vulnerability to interference-related spatial memory deficits becomes more apparent with age in this model. Although a significant overall effect of age on plaque burden was detected, it did not resolve into significant pairwise regional differences after post-hoc test, and plaque burden did not correlate with memory performance, suggesting that the age-related behavioral vulnerability is not straightforwardly explained by regional amyloid load alone. Importantly, we should note that these subgroup analyses involved smaller sample sizes than the pooled comparisons and should therefore be interpreted as informative but not definitive.

### A*β* pathology, c-Fos mapping, and behavioral performance

We quantified Aβ plaque burden across hippocampal and entorhinal regions to place the behavioral findings in a pathological context. Plaque density was highest in the dentate gyrus, consistent with prior descriptions of regional amyloid distribution in this model (Radde et al., 2006). However, plaque burden did not correlate significantly with memory performance in either non-competing or competing comparisons. This result adds to a broader literature indicating that cognitive impairment is not always linearly predicted by plaque load and supports the view that amyloidosis may influence behavior through broader synaptic and circuit-level consequences (Viana da Silva et al., 2024) or neuroinflammatory processes (Heneka et al., 2015) rather than plaque burden alone.

The hippocampus and EC are critical for spatial learning and memory, with CA3 supporting pattern separation and retrieval (Leutgeb et al., 2007; Rolls, 2013) and CA1 integrating and updating spatial representations, particularly under conditions where interference or competition between memories occurs (Deshmukh, 2021). To relate behavior to regional neuronal recruitment, we used c-Fos immunolabeling after novelty exposure on the day following OUL testing. These data should be interpreted as a proxy of recent neuronal recruitment rather than as a direct measure of neuronal firing, hyperactivity, or synaptic plasticity. At the group level, c-Fos-positive cell density did not differ significantly between genotypes in most hippocampal or entorhinal regions, although there was a trend toward lower recruitment in DG. In control mice, higher CA3 c-Fos expression after novelty exposure was associated with better performance in the non-competing object-displacement comparison, whereas this relationship was absent in APP/PS1 mice. This pattern is consistent with the established role of CA3 in pattern completion and retrieval of stored representations (Rolls, 2013; Guzman et al., 2016).

The subgroup c-Fos analyses also suggested reduced DG recruitment in APP/PS1 females and increased mEC layer II recruitment in younger APP/PS1 mice. These observations are potentially interesting in light of prior work showing early hyperexcitability (Busche et al., 2012; Targa Dias Anastacio et al., 2022) or altered activity recruitment in entorhinal-hippocampal circuits in AD models and patients (Celone et al., 2006; Kazim et al., 2017; Targa Dias Anastacio et al., 2022), as well as studies reporting reduced hippocampal recruitment in APP/PS1 mice during behavior (Cayzac et al., 2015; Poll et al., 2020). Likewise, the reduced DG c-Fos signal observed in APP/PS1 females may be consistent with reports that DG recruitment changes dynamically over time and with experience (Lamothe-Molina et al., 2022). The differences we found between sexes are consistent with earlier studies indicating different neural circuit engagement during memory processing between males and females (Florido et al., 2024). However, the present study cannot determine whether these differences reflect compensatory recruitment, altered excitability, or other forms of circuit adaptation. C-Fos mapping provides only a limited temporal snapshot following novelty exposure and cannot resolve activity dynamics during the OUL task itself. For that reason, we discuss these findings as correlational and hypothesis-generating rather than mechanistic.

Finally, given prior evidence that CA1 contributes to resolving overlapping or interfering memories (Dimsdale-Zucker et al., 2018; Gonzalez et al., 2019; Zou et al., 2023), we also examined whether CA1 c-Fos expression related to performance under competing conditions. In control mice, CA1 c-Fos expression following novelty exposure was significantly associated with performance in one of the two competing comparisons (Novel vs. Original #2), whereas no such association was found in APP/PS1 mice. This is consistent with the proposed role of CA1 as a comparator between stored and current spatial representations, a function thought to be critical for detecting and resolving interference between overlapping memory traces (Dimsdale-Zucker et al., 2018; Gonzalez et al., 2019). Notably, the association was specific to the comparison involving Original #2, the object carrying overlapping spatial history, because its previously learned coordinates after displacement coincide with Updated object location. While Original #1 occupies a distinct spatial location, the shared coordinates of Original #2 and the Updated object create a high-interference condition. CA1 must therefore function as a comparator to resolve the conflict between the stored representation of Original #2 and the current sensory input of the Updated object, a process that appears specifically disrupted in the APP/PS1 genotype. The absence of this relationship in APP/PS1 mice suggests that the functional coupling between CA1 recruitment and competing memory performance may be disrupted under amyloidosis. That the association was absent for the Novel vs. Updated comparison in both genotypes may reflect different mnemonic demands, as the updated location carries a more recent but weaker trace rather than two overlapping well-established representations.

Taken together, the c-Fos findings suggest that regional and sex-dependent differences in neuronal recruitment following novelty exposure may contribute to the behavioral variability observed in this model. The reduced c-Fos expression we found in the DG in female APP/PS1 mice is important in light of previous work identifying that DG activation dynamically changes across days, whereas CA1 activation remains more stable (Lamothe-Molina et al., 2022). This provides a potential mechanism for the impaired interference handling, while explaining the preserved single-object recall in APP/PS1 mice. The differences we found between sexes are in line with earlier studies that indicate different neural circuit engagement during memory processing between males and females (Florido et al., 2024).

### Limitations of the study

While our study provides novel insights into spatial memory deficits in APP/PS1 mice, several limitations should be acknowledged. First, our findings are correlative, preventing direct causal conclusions. Future studies using optogenetic or chemogenetic manipulations could clarify the specific contributions of CA3 and CA1 to spatial interference resolution and memory updating. Incorporating additional behavioral paradigms, such as temporal order memory tasks, could provide a more comprehensive assessment of cognitive decline in APP/PS1 mice. Second, although the OUL paradigm captures an important aspect of spatial memory updating, additional behavioral assays, including tasks probing temporal order or other forms of flexible memory, would help determine how generalizable these effects are across cognitive domains. Finally, while c-Fos mapping provides useful information about recent neuronal recruitment following novelty exposure, it offers only a limited snapshot of brain activity and cannot resolve the temporal dynamics or functional properties of the underlying circuits.

### Implications for Alzheimer’s disease research

Within these constraints, the present findings support the view that memory updating under interference is a sensitive behavioral domain in this preclinical AD model. The OUL paradigm complements more widely used rodent memory tasks by probing not only whether an animal detects spatial novelty, but also how it handles competition between older and newer spatial information. That feature may be especially relevant for studying cognitive change in disorders in which memory is not simply lost, but becomes more difficult to use flexibly across changing contexts. Our results identify a behavioral phenotype that may be useful for testing hypotheses about early hippocampal-entorhinal dysfunction, age-related vulnerability, and the relationship between amyloidosis and flexible memory use. Future work combining OUL-like paradigms with circuit-level manipulations or longitudinal measures of neuronal activity may help clarify how interference-related memory deficits emerge and whether they can serve as an informative preclinical outcome measure.

## Significance statement

Using the OUL paradigm, we show that amyloidosis in APP/PS1 mice is associated with less reliable spatial memory performance and weaker handling of competing spatial information, highlighting memory updating under interference as a sensitive behavioral domain in preclinical AD.

## Data Availability

The raw data supporting the conclusions of this article will be made available by the authors, without undue reservation.
