# Supplementary material for: Age-dependent vulnerability to spatial memory interference in APP/PS1 mice
Source: Front Aging Neurosci. 2026 Jun 2;18:1794153. doi: 10.3389/fnagi.2026.1794153 (PMC13269207; doi:10.3389/fnagi.2026.1794153)
Supplement: Supplementary file 1 [file Supplementary_file_1.docx]

**Supplemental Information**

for manuscript *Age-Dependent Vulnerability to Spatial Memory Interference in APP/PS1 Mice*

by D. Mitrovic-Tartanoglu et al.

**Supplementary Figures**

**
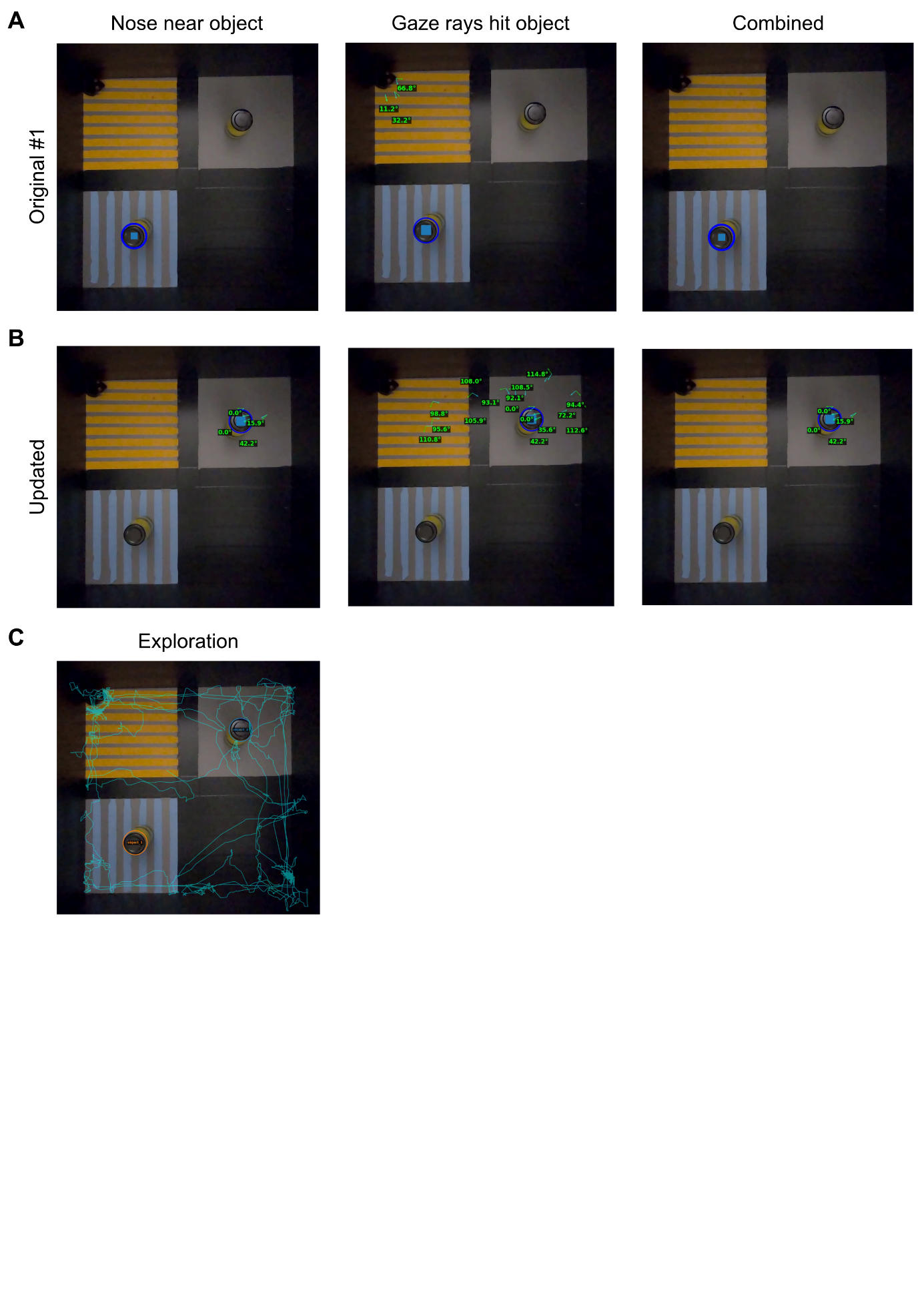
**

**Supplementary Figure 1.** **Visual validation of automated detection of object exploration events. Representative frames illustrating automated identification of object interaction events, including gaze direction and path trace overlays.** (A) Visualization of exploration criteria for an object in Original #1 location. Frames show instances in which the mouse’s nose is within the defined proximity threshold (left), gaze orientation toward the nearest point of the object (middle), and detection of exploration events when both criteria are satisfied (right). (B) Same as in A, only for object in Updated location. (C) Path trace during the period required to reach 10 s of total object exploration.

**
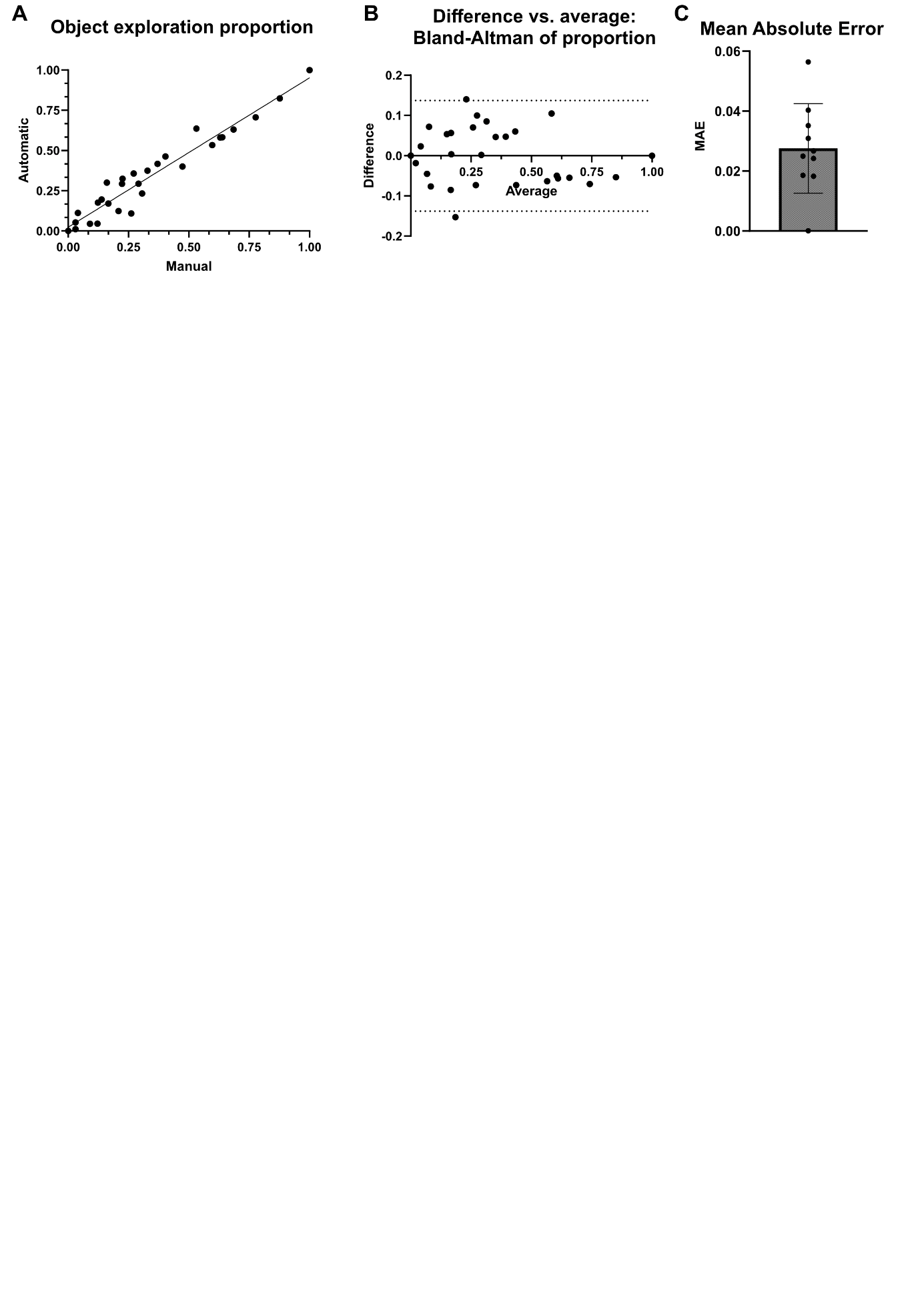
**

**Supplementary Figure 2.** **Automatic scoring assessment in comparison to manual scoring. (A)** scoring across all observations with linear regression. **(B)** Bland–Altman plot showing the difference between automated and manual scoring as a function of their mean. The solid line indicates the mean difference (bias), and dashed lines represent the 95% limits of agreement. Each point corresponds to a single object observation. **(C)** Mean absolute error (MAE) in object-wise exploration proportions calculated per mouse and session. Each dot represents one mouse-session, indicating the average absolute difference between manual and automated scoring across objects. See Supplementary Table 11 and 12 for more statistical details and individual values.


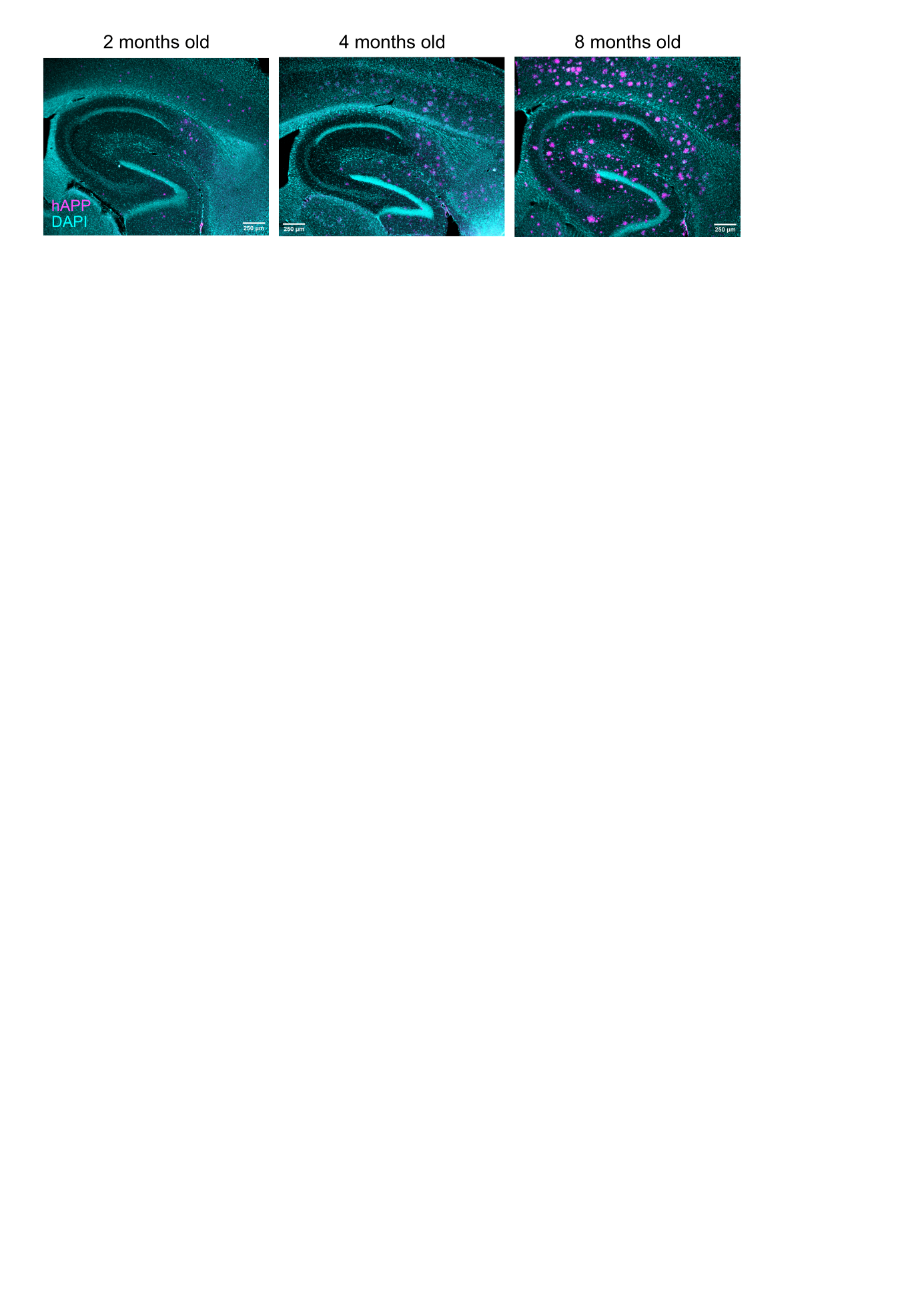


**Supplementary Figure 3. Aβ pathology progression across different ages.** Immunostaining of sagittal brain sections stained for hAPP (magenta) and with DAPI (cyan), depicting hippocampus of APP/PS1 mice for ages of 2-, 4- and 8-months-old. hAPP, human amyloid precursor protein.


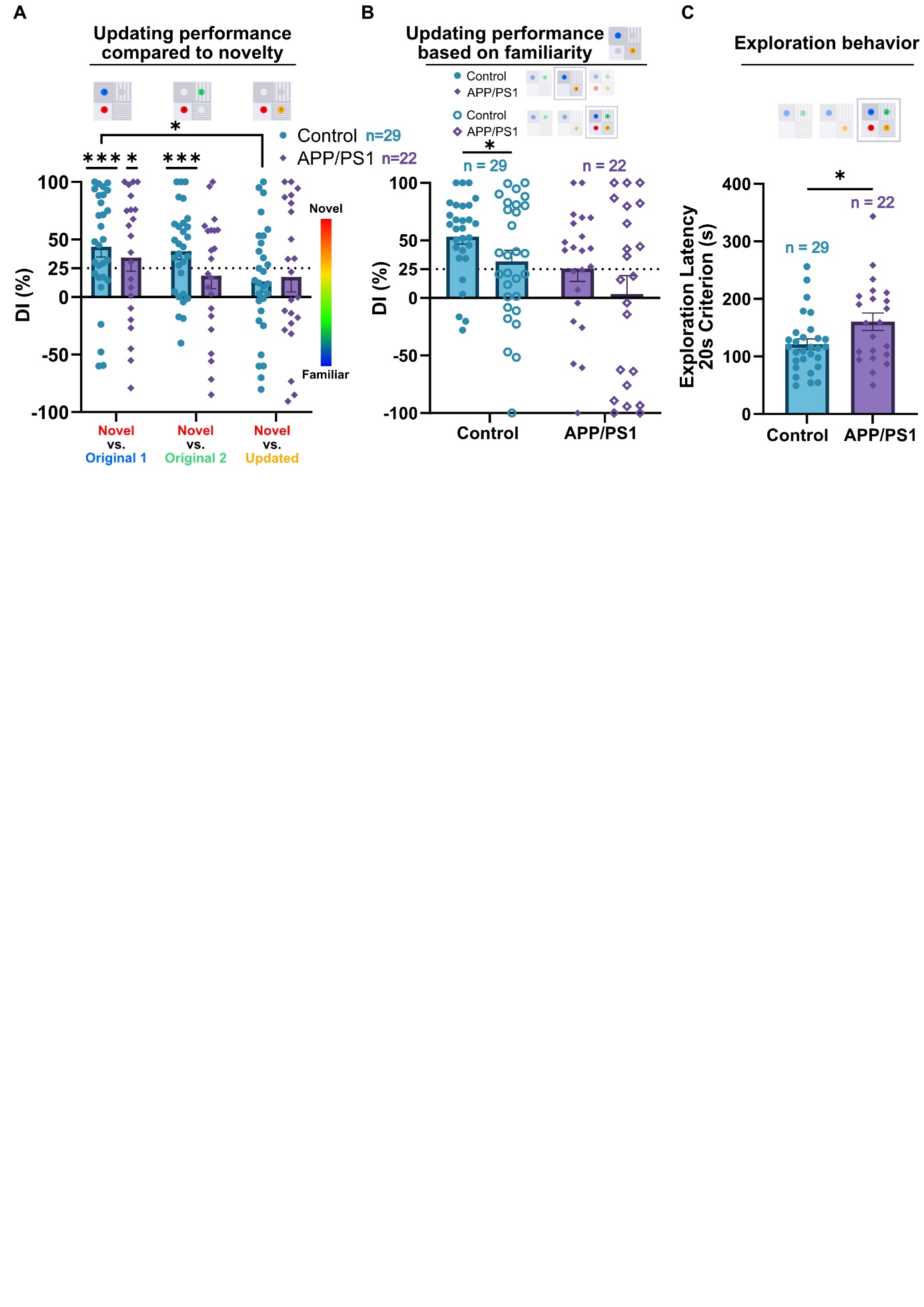


**Supplementary Figure 4. Updating performance and exploration behavior without exclusion based on object displacement memory performance.** **(A)** Discrimination index (DI) during the updating performance test based on novelty across three memory type comparisons (Novel vs. Original 1, Novel vs. Original 2, Novel vs. Updated) in control (n=29) and APP/PS1 (n=22) mice. Memory type differences (p=0.032, ANOVA; control: novel-O1 vs. novel-updated, p=0.022, Tukey's post-hoc), genotype (p=0.381). **(B)** DI during the updating performance test based on familiarity in control (n=29) and APP/PS1 (n=22) mice (control: p=0.031; APP/PS1: p=0.176, Wilcoxon matched-pairs signed rank test). **(C)** Exploration latency to reach the 20-second criterion during the updating performance session (p=0.048, MW). Data are presented as mean ± SEM. *p ≤ 0.05, **p ≤ 0.01, ***p ≤ 0.001. See Supplementary Table 13 for more statistical details.

**
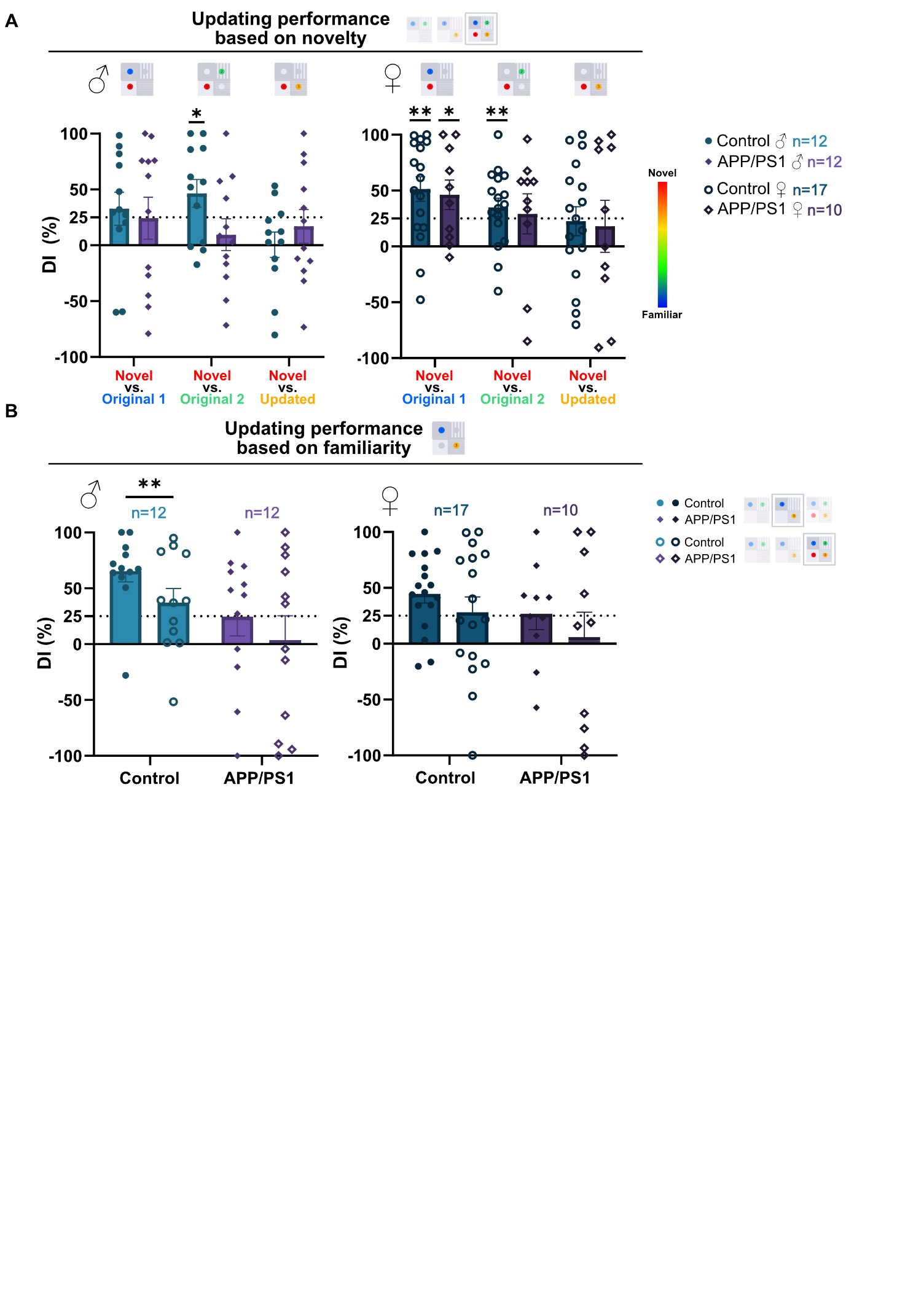
**

**Supplementary Figure 5. Updating performance based on novelty and familiarity without exclusion based on object displacement memory performance, with respect to sex. (A)** DI during the updating performance test based on novelty across three memory type comparisons (Novel vs. Original 1, Novel vs. Original 2, Novel vs. Updated) in male (control n=12, APP/PS1 n=12) and female (control n=17, APP/PS1 n=10) mice. Males: memory type (p=0.247, ANOVA), genotype (p=0.497). Females: memory type (p=0.077, ANOVA), genotype (p=0.731). **(B)** DI during the updating performance test based on familiarity in male (control n=12, APP/PS1 n=12) and female (control n=17, APP/PS1 n=10) mice. Males: control p=0.005, APP/PS1 p=0.266; females: control p=0.863, APP/PS1 p=0.863 (Wilcoxon matched-pairs signed rank test, HB post-hoc correction). Data are presented as mean ± SEM. *p ≤ 0.05, **p ≤ 0.01. See Supplementary Table 14 for more statistical details.

**
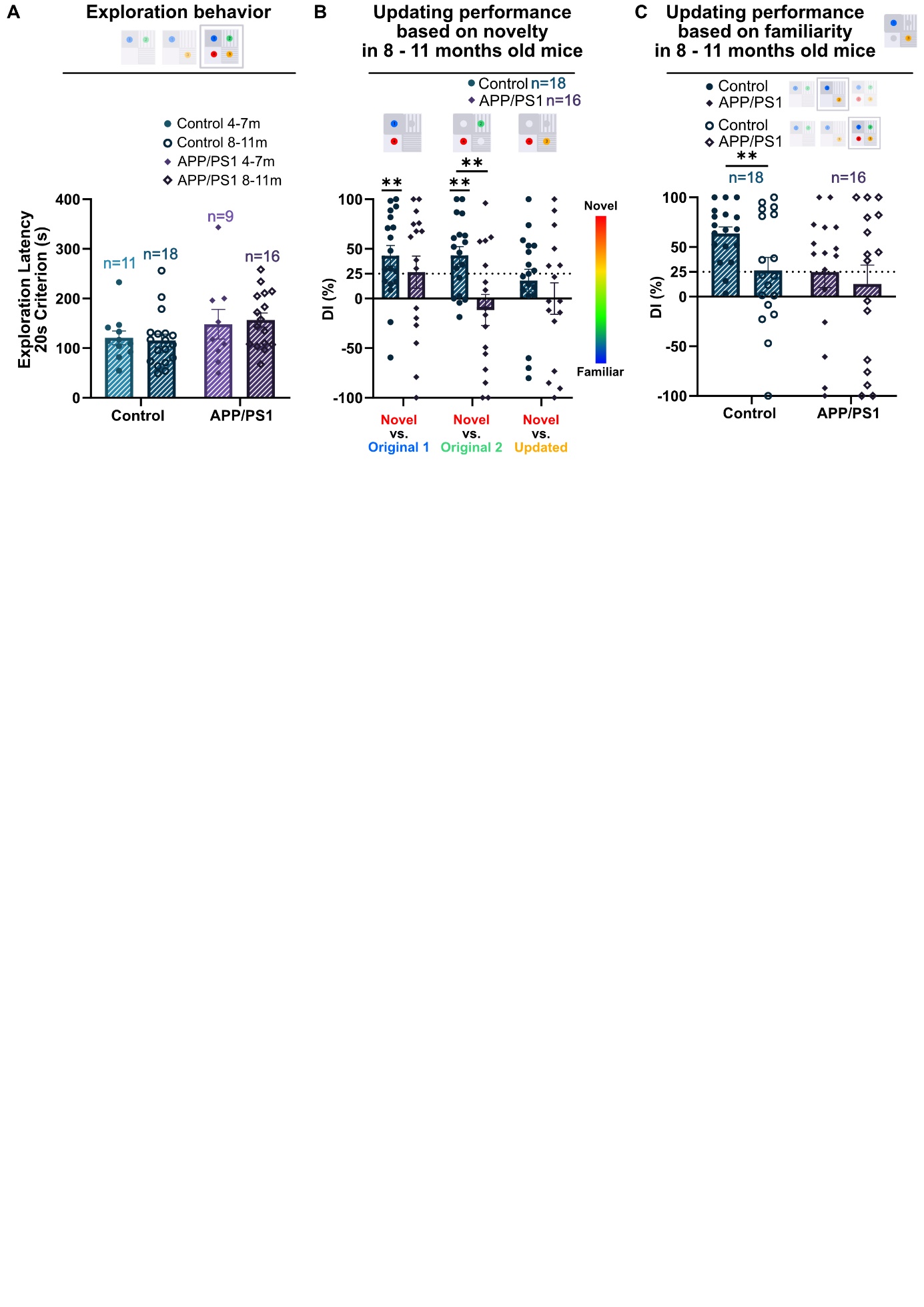
**

**Supplementary Figure 6. Updating performance based on novelty and familiarity and exploration behavior without exclusion based on object displacement memory performance, in older mice.**  **(A)** Exploration latency to reach the 20-second criterion during the updating performance session in 4-7-month-old control (n=11) and APP/PS1 (n=9) mice (p=0.656, MW) and in 8-11-month-old control (n=18) and APP/PS1 (n=16) mice (p=0.065). **(B)** DI during the updating performance test based on novelty across three memory type comparisons (Novel vs. Original #1, Novel vs. Original #2, Novel vs. Updated) in 8-11-month-old control (n=18) and APP/PS1 (n=16) mice. Memory type (p=0.085, ANOVA), genotype (p=0.025; ANOVA, Novel vs. Original #2: p=0.005, Tukey's post-hoc). **(C)** DI during the updating performance test based on familiarity in 8-11-month-old control (n=18) and APP/PS1 (n=16) mice (control: p=0.006; APP/PS1: p=0.720, Wilcoxon matched-pairs signed rank test, HB post-hoc correction). Data are presented as mean ± SEM. *p ≤ 0.05, **p ≤ 0.01. See Supplementary Table 15 for more statistical details.

**
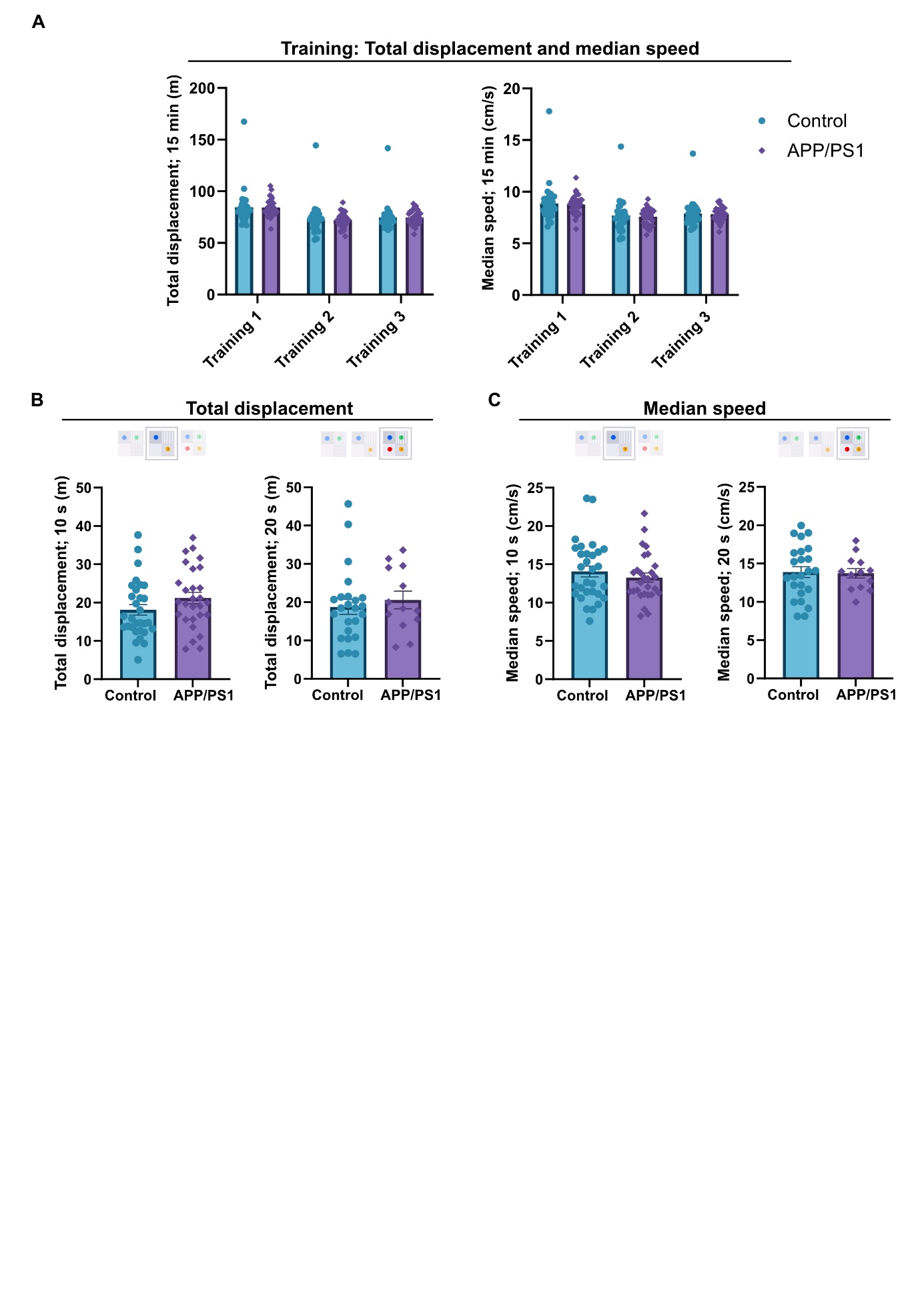
**

**Supplementary Figure 7. Exploration parameters during OUL paradigm.** **(A)** Total displacement (left) and median speed (right) during the whole training sessions (15 min each). **(B)** Total displacement during object displacement and updating performance (during exploration until mice reached 10 and 20 seconds in total of object exploration during object displacement and updating performance respectively). **(C)** Same as in B, only showing median speed. No significant differences were detected between the genotypes. See Supplementary Table 16 for more statistical details.


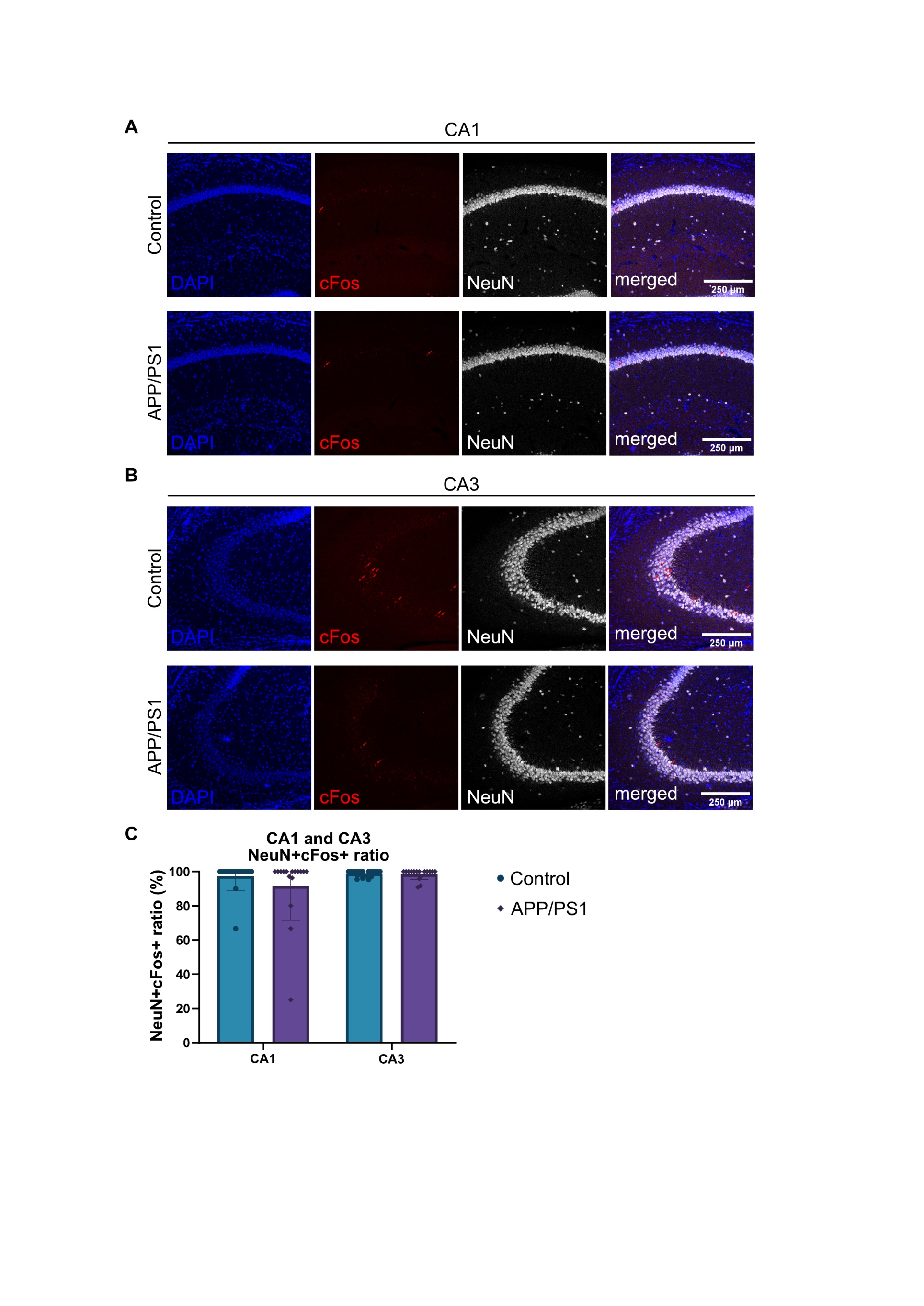


**Supplementary Figure 8. NeuN+cFos+ vs. NeuN-cFos+ cell distribution in CA1 and CA3.** **(A)** Immunohistochemistry staining examples for CA1 and CA3 with DAPI, cFos and NeuN markers. **(B)** NeuN+cFos+ vs. NeuN-cFos+ cell distribution in CA1 and CA3. See Supplementary Table 17 for more statistical details.

**
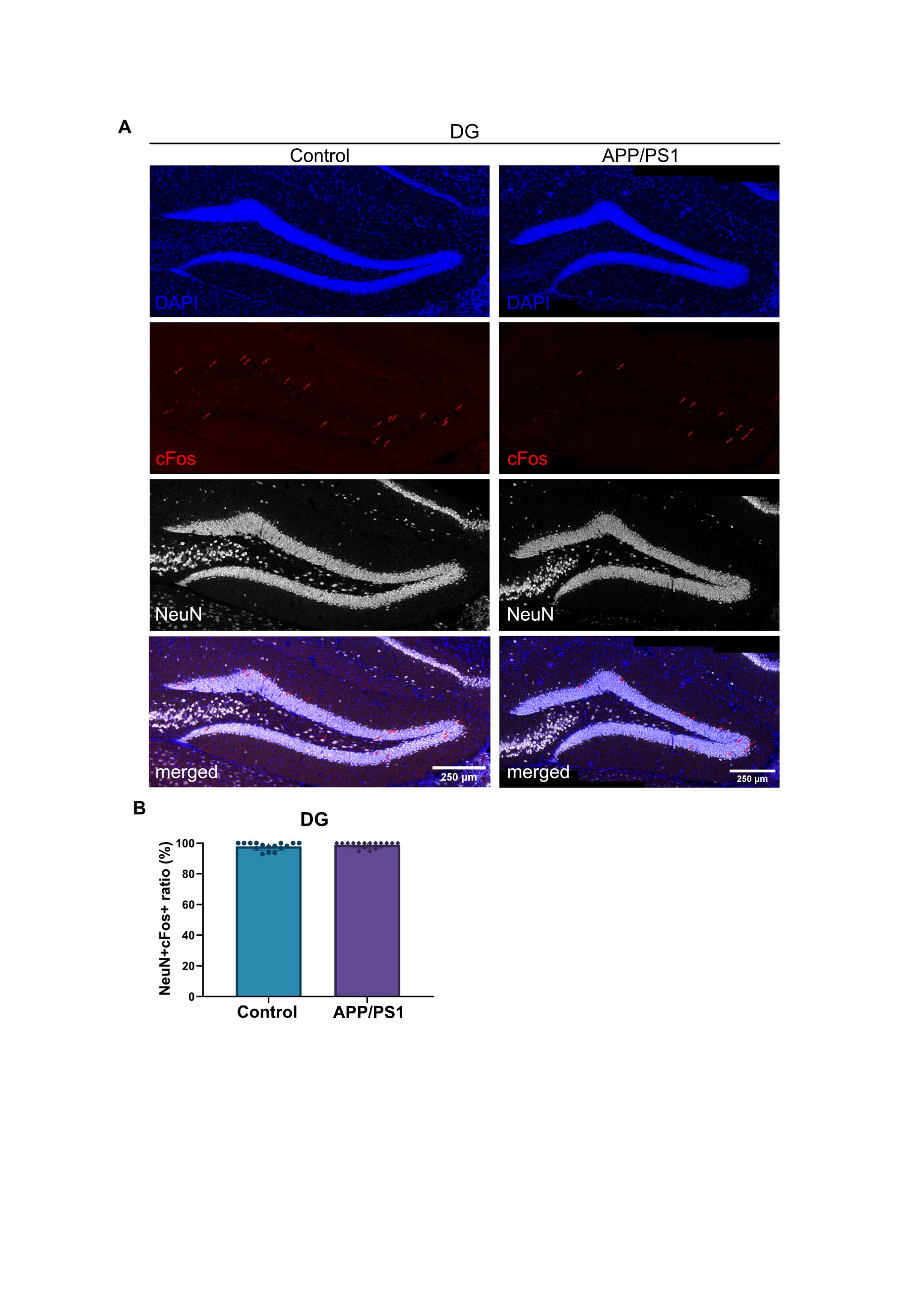
**

**Supplementary Figure 9.** **NeuN+cFos+ vs. NeuN-cFos+ cell distribution in DG.** **(A)** Immunohistochemistry staining examples for DG with DAPI, cFos and NeuN markers. **(B)** NeuN+cFos+ vs. NeuN-cFos+ cell distribution in DG. See Supplementary Table 17 for more statistical details.

**
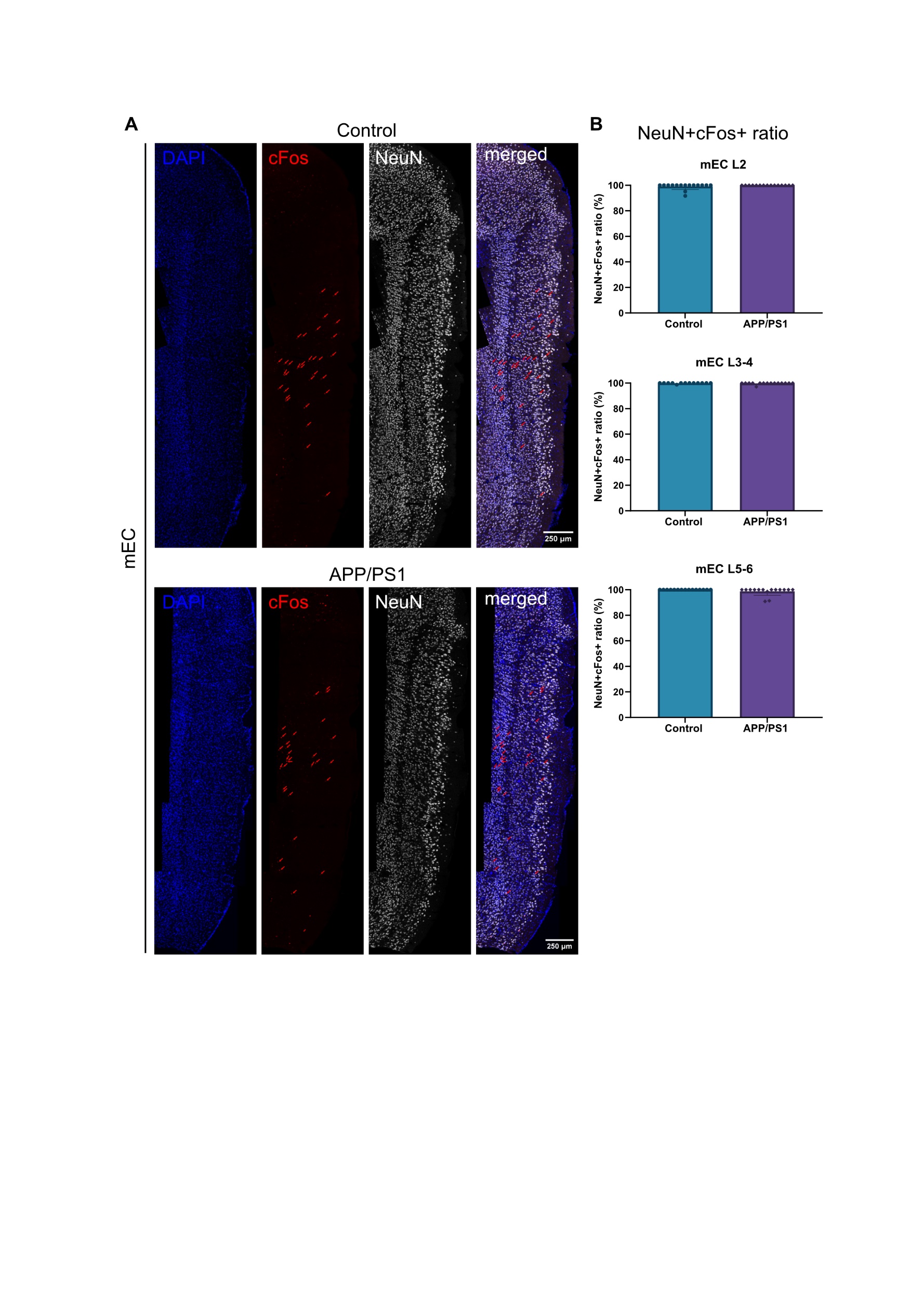
**

**Supplementary Figure 10.** **NeuN+cFos+ vs. NeuN-cFos+ cell distribution in mEC layers 2, 3-4 and 5-6.** **(A)** Immunohistochemistry staining examples for mEC with DAPI, cFos and NeuN markers. **(B)** NeuN+cFos+ vs. NeuN-cFos+ cell distribution in mEC layers 2, 3-4 and 5-6. See Supplementary Table 17 for more statistical details.

**
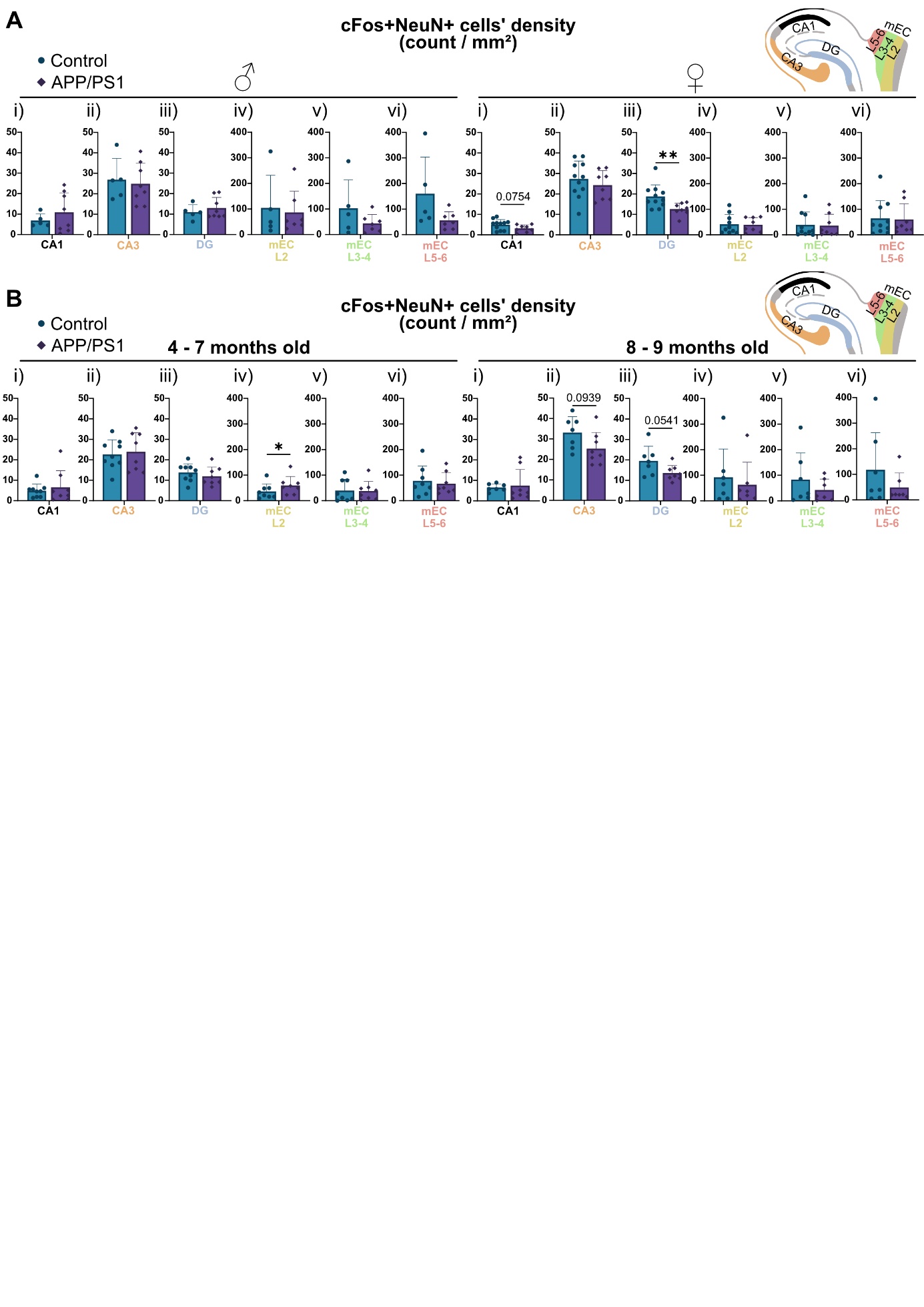
Supplementary Figure 11. Sex- and age-dependent c-Fos differences in APP/PS1 mice. (A)** c-Fos+NeuN+ cells’ density in different sex groups (left: males; right: females). Significant difference in cFos+ neuron density in DG between control (n=11) and APP/PS1 (n=8) female mice (p_DG_=0.0036, MW). **(B)** Similar to A, split into different age groups; 4-7 and 8-9-months-old mice. Significant difference in cFos+ neuron density in mEC layer 2 between young control (n=8) and APP/PS (n=8) mice (p_mECL2_=0.0499, MW). Data are presented as mean ± SEM. *p ≤ 0.05. CA, cornu ammonis; DG, dentate gyrus; HC, hippocampus; mEC, medial entorhinal cortex; DI, discrimination index. See Supplementary Table 20 for more statistical details.

**Supplementary Tables**

**Supplementary Table 1.** Key resources

| Reagent/Resource | Source | Identifier |
| --- | --- | --- |
| Antibodies | | |
| Mouse IgG1 Purified anti-β-Amyloid, 1-16 Antibody (6E10 clone) | BioLegend | Cat# 803001 |
| Chicken Anti-NeuN Polyclonal Antibody | Sigma-Aldrich | Cat# ABN91 |
| Guinea pig Anti-GFAP Polyclonal Antibody | Sysy | Cat# 173004 |
| Rabbit IgG Anti-cFos Monoclonal Antibody | Cell Signalling Technology | Cat# 2250S |
| Alexa Fluor™ 488 (anti-Mouse) | Fisher Scientific | Cat# A32723 |
| Alexa Fluor™ 488 (anti-chicken) | Thermo Fisher | Cat# A11039 |
| Alexa Fluor™ 555 (anti-rabbit) | Thermo Fisher | Cat# A32732 |
| Alexa Fluor™ 647 (anti-guinea pig) | Thermo Fisher | Cat# A21450 |
| Other reagents | | |
| PBS (10x), pH 7.4 | Thermo Fisher | Cat# 70011044 |
| Paraformaldehyde (PFA) Solution, 4% in PBS | Thermo Fisher | Cat# J19943.K2 |
| Prolong Gold Antifade | Thermo Fisher | Cat# P36930 |
| Experimental models | | |
| B6.Cg-Tg(Thy1-APPSw,Thy1-PSEN1*L166P)21Jckr | Matthias Jucker |  |
| C57BL/6J | Charité FEM |  |

**Supplementary Table 2.** Genotype and sex of experimental mice

| **Experimental group** | **n**  **(total nr. of mice)** | **n**  **(male mice)** | **n**  **(female mice)** | **n**  **(4-7 months old)** | **n**  **(8-11 months old)** |
| --- | --- | --- | --- | --- | --- |
| Training / Habituation session | | | | | |
| control | 34 | 16 | 18 | 13 | 21 |
| APP/PS1 | 34 | 18 | 16 | 12 | 22 |
| Object displacement session* | | | | | |
| control | 30 | 13 | 17 | 11 | 19 |
| APP/PS1 | 28 | 14 | 14 | 9 | 19 |
| Updating performance session* | | | | | |
| control | 25 | 12 | 13 | 8 | 17 |
| APP/PS1 | 16 | 9 | 7 | 4 | 12 |

* Fluctuations in n reflect the exclusion of sessions where mice failed to meet the minimum exploration threshold or lack of specific objects exploration, preventing the calculation of the Discrimination Index (division by zero).

**Supplementary Table 3.** DeepLabCut network evaluation values

| **Metric** | **Values** |
| --- | --- |
| **Train error (px)** | 1.74 |
| **Test error (px)** | 20.36 |
| **Test error w/ p-cutoff (px)** | 8.93 |
| **Test error w/ p-cutoff (mm)** | 6.43 |

**Supplementary Table 4.** Statistics for Aβ pathology – Fig. 1, Fig. 3, Fig. 4 and Fig. 5

| **Fig. panel** | **Graph descript-ion** | **n**  **(mice)**  **CA1** | | **n (mice)**  **CA3** | | **n**  **(mice)**  **DG** | | **n**  **(mice)**  **mEC** | | **Stat.**  **test** | **Stat. metrics** | **P value** | **Adjusted P value** | | **Sig.** |
| --- | --- | --- | --- | --- | --- | --- | --- | --- | --- | --- | --- | --- | --- | --- | --- |
| **Fig. 1B** | Aβ plaque density  (pooled) | 26 | | 26 | | 26 | | 24 | | KW with Dunn’s post-hoc | KW=  33.91 | <0.0001 | CA1 vs. CA3 | >0.9999 | ns |
|  |  |  |  |  |  |  |  |  |  |  |  |  | CA1 vs. DG | <0.0001 | **** |
|  |  |  |  |  |  |  |  |  |  |  |  |  | CA1 vs. mEC | >0.9999 | ns |
|  |  |  |  |  |  |  |  |  |  |  |  |  | CA3 vs. DG | <0.0001 | **** |
|  |  |  |  |  |  |  |  |  |  |  |  |  | CA3 vs. mEC | 0.5510 | ns |
|  |  |  |  |  |  |  |  |  |  |  |  |  | DG vs. mEC | 0.0036 | ** |
| **Fig. 3A** | Aβ plaque density  (sex) | M | F | M | F | M | F | M | F | Two-way ANOVA  with B post-hoc | Sex:  F (1, 94) = 8.404 | 0.0047 | CA1 | >0.9999 | ns |
|  |  | 13 | 13 | 13 | 13 | 13 | 13 | 12 | 12 |  |  |  | CA3 | 0.2955 | ns |
|  |  |  |  |  |  |  |  |  |  |  |  |  | DG | 0.2214 | ns |
|  |  |  |  |  |  |  |  |  |  |  |  |  | mEC | 0.2463 | ns |
| **Fig. 4A** | Aβ plaque density  (age) | 4-7 m | 8-9 m | 4-7 m | 8-9 m | 4-7 m | 8-9 m | 4-7 m | 8-9 m | Two-way ANOVA  with B post-hoc | Age:  F (1, 94) = 8.584 | 0.0043 | CA1 | 0.5786 | ns |
|  |  | 10 | 16 | 10 | 16 | 10 | 16 | 12 | 12 |  |  |  | CA3 | 0.7002 | ns |
|  |  |  |  |  |  |  |  |  |  |  |  |  | DG | 0.5562 | ns |
|  |  |  |  |  |  |  |  |  |  |  |  |  | mEC | 0.5180 | ns |
| **Fig. 5C**  **(top)** | Corr. with memory perform-  ance:  Novel vs. O1 | CA1 | | CA3 | | DG | | mEC | | Spear-man corr. & simple linear reg. | CA1 r_s_= 0.1581 | 0.6621 | - | | ns |
|  |  |  |  |  |  |  |  |  |  |  | CA1 r^2^=0.0003 | 0.9645 |  |  | ns |
|  |  | 10 | | 10 | | 8 | | 6 | |  | CA3 r_s_=0.1108 | 0.7604 | - | | ns |
|  |  |  |  |  |  |  |  |  |  |  | CA3 r^2^=0.0105 | 0.7779 |  |  | ns |
|  |  |  |  |  |  |  |  |  |  |  | DG r_s_= 0.2036 | 0.6294 | - | | ns |
|  |  |  |  |  |  |  |  |  |  |  | DG r^2^=0.0841 | 0.4859 |  |  | ns |
|  |  |  |  |  |  |  |  |  |  |  | mEC r_s_=0.0286 | >0.9999 | - | | ns |
|  |  |  |  |  |  |  |  |  |  |  | mEC r^2^=0.0063 | 0.8811 |  |  | ns |
| **Fig. 5C**  **(mid)** | Corr. with memory perform-  ance:  Novel vs. O2 | CA1 | | CA3 | | DG | | mEC | | Spear-man corr. & simple linear reg. | CA1 r_s_=-0.224 | 0.5367 | - | | ns |
|  |  |  |  |  |  |  |  |  |  |  | CA1 r^2^=0.085 | 0.4131 |  |  | ns |
|  |  | 10 | | 10 | | 8 | | 6 | |  | CA3 r_s_=-0.620 | 0.0634 | - | | ns |
|  |  |  |  |  |  |  |  |  |  |  | CA3 r^2^=0.321 | 0.0876 |  |  | ns |
|  |  |  |  |  |  |  |  |  |  |  | DG r_s_=-0.024 | 0.9768 | - | | ns |
|  |  |  |  |  |  |  |  |  |  |  | DG r^2^=0.002 | 0.9131 |  |  | ns |
|  |  |  |  |  |  |  |  |  |  |  | mEC r_s_=-0.371 | 0.4972 | - | | ns |
|  |  |  |  |  |  |  |  |  |  |  | mEC r^2^=0.2829 | 0.2774 |  |  | ns |
| **Fig. 5C**  **(bott-om)** | Corr. with memory perform-  ance:  Novel vs. updated | CA1 | | CA3 | | DG | | mEC | | Spear-man corr. & simple linear reg. | CA1 r_s_=-0.224 | 0.5367 | - | | ns |
|  |  |  |  |  |  |  |  |  |  |  | CA1 r^2^=0.015 | 0.7394 |  |  | ns |
|  |  | 10 | | 10 | | 8 | | 6 | |  | CA3 r_s_=-0.385 | 0.2432 | - | | ns |
|  |  |  |  |  |  |  |  |  |  |  | CA3 r^2^=0.1537 | 0.2330 |  |  | ns |
|  |  |  |  |  |  |  |  |  |  |  | DG r_s_=-0.262 | 0.5364 | - | | ns |
|  |  |  |  |  |  |  |  |  |  |  | DG r^2^=0.191 | 0.2794 |  |  | ns |
|  |  |  |  |  |  |  |  |  |  |  | mEC r_s_=-0.714 | 0.1361 | - | | ns |
|  |  |  |  |  |  |  |  |  |  |  | mEC r^2^=0.5809 | 0.0781 |  |  | ns |

Abbreviations: M, male; F, female; Corr., correlation; KW, Kruskal Wallis; B, Bonferroni; MW, Mann-Whitney U; HB, Holm-Bonferroni; CA, Cornu Ammonis; DG, dentate gyrus; mEC, medial entorhinal cortex

**Supplementary Table 5.** Statistics for memory performance (DI) – Fig. 1

| **Fig. panel** | **Graph description** | **n**  **(ctrl)** | **n**  **(tg)** | **Statistical**  **test** | **Stat. metrics** | | **P value** | **Adjusted P value** | **Sig.** |
| --- | --- | --- | --- | --- | --- | --- | --- | --- | --- |
| **Fig. 1E** | Object displacement | 30 | 28 | One sample Wilcoxon test (theoretical mean: 0) | **ctrl** | W = 441.0 median = 61.66 | <0.0001 | - | *** |
|  |  |  |  |  | **tg** | W = 161.0 median = 34.16 | 0.0671 | - | ns |
|  |  |  |  | MW  (ctrl vs. tg) | MW-U: 264.5 | | 0.0149 | - | * |
| **Fig. 1G** | Updating performance (novel vs. O1, O2, updated) | 24 | 13 | One sample Wilcoxon test (theoretical mean: 0) with HB post-hoc | O1 | ctrl: W = 265.0, median = 48.09 | 0.0002 | 0.0004 | *** |
|  |  |  |  |  |  | tg: W = 90.0, median = 74.97 | 0.0322 | 0.1288 | ns |
|  |  |  |  |  | O2 | ctrl: W = 233.0 , median = 35.42 | <0.0001 | 0.0003 | *** |
|  |  |  |  |  |  | tg: W = 20.0, median = 12.46 | 0.8394 | >0.9999 | ns |
|  |  |  |  |  | updated | ctrl: W = 99.00 , median = 12.20 | 0.3029 | 0.3029 | ns |
|  |  |  |  |  |  | tg: W = -1.0, median =-12.96 | 0.5879 | >0.9999 | ns |
|  |  |  |  | Two-way ANOVA with Tukey’s post-hoc | GT | F (1, 35) = 2.243 | 0.1432 | - | ns |
|  |  |  |  |  | Memory type* | F (1.993, 69.75) = 8.751 | 0.0004 | Control:  Novel-O1 vs. novel-updated  0.0216 | * |
|  |  |  |  |  |  |  |  | APP/PS1:  Novel-O1 vs. novel-updated  0.0319 | * |
| **Fig. 1H** | Updating performance (updated vs. O1) | 24 | 13 | Wilcoxon matched-pairs signed rank test | ctrl | W= -198.0 | 0.0035 | - | ** |
|  |  |  |  |  | tg | W=-29.0 | 0.3396 |  | ns |

*only significant post-hoc results shown

Abbreviations: O1-2, Original 1-2; ctrl, control; tg, transgenic; HB, Holm-Bonferroni; MW, Mann-Whitney U; GT, genotype; Upd., updated

**Supplementary Table 6.** Binomial test analysis (control vs. APP/PS1) for memory performance – Fig. 1, Fig. 3 and Fig. 4

| **Fig. panel** | **Graph description** | **n**  **(control/**  **expected)** | **n**  **(APP/PS1/observed)** | **% DI ≥ 25**  **(control/**  **expected)** | **% DI ≥ 25**  **(APP/PS1/observed)** | **Stat.**  **test** | **P value** | **Sig.** |
| --- | --- | --- | --- | --- | --- | --- | --- | --- |
| **Fig. 1F** | Object displacement  -  pooled | 30 | 28 | 83.33 % | 57.14 % | Binomial test | One-tailed: 0.0010  Two-tailed: 0.0010 | *** |
| **Fig. 3C Males**  **(top)** | Object displacement  -  males | 13 | 14 | 92.31 % | 64.29 % | Binomial test | One-tailed: 0.0030  Two-tailed: 0.0030 | ** |
| **Fig. 3C Females**  **(bottom)** | Object displacement  -  females | 17 | 14 | 76.47 % | 50.00 % | Binomial test | One-tailed: 0.0280  Two-tailed: 0.0280 | * |
| **Fig. 4D**  **4 – 7 months**  **(top)** | Object displacement  -  4 – 7 months | 11 | 9 | 72.73 % | 44.44 % | Binomial test | One-tailed: 0.0688  Two-tailed: 0.0688 | ns |
| **Fig. 4D**  **8 – 11 months**  **(bottom)** | Object displacement  -  8 – 11 months | 19 | 19 | 89.47 % | 63.16 % | Binomial test | One-tailed: 0.0023  Two-tailed: 0.0023 | ** |

**Supplementary Table 7.** Statistics for exploration behavior and correlation to memory performance – Fig. 2

| **Fig. panel** | **Graph description** | **n**  **(ctrl)** | **n**  **(tg)** | **Statistical**  **test** | **Stat. metrics** | | **P value** | **Sig.** |
| --- | --- | --- | --- | --- | --- | --- | --- | --- |
| **Fig. 2A (top)** | Exploration latency: object displacem. | 30 | 28 | MW  (ctrl vs. tg) | MW-U= 295 | | 0.0522 | ns |
| **Fig. 2A (bottom)** | Exploration latency: updating perform. | 24 | 13 | MW  (ctrl vs. tg) | MW-U= 123 | | 0.3056 | ns |
| **Fig. 2B (left)** | Corr. DI with exploration behavior:  Update vs. O1 | 30 | 28 | Spearman correlation & simple linear regression | ctrl | r_s_=0.04474 | 0.8144 | ns |
|  |  |  |  |  |  | r^2^=0.0139 | 0.5350 | ns |
|  |  |  |  |  | tg | r_s_=-0.00931 | 0.9625 | ns |
|  |  |  |  |  |  | r^2^=0.0012 | 0.8605 | ns |
| **Fig. 2B (right)** | Corr. DI with exploration behavior:  Novel vs. O1 | 24 | 13 | Spearman correlation & simple linear regression | ctrl | r_s_=-0.2165 | 0.3095 | ns |
|  |  |  |  |  |  | r^2^=0.0205 | 0.5044 | ns |
|  |  |  |  |  | tg | r_s_=-0.2531 | 0.4007 | ns |
|  |  |  |  |  |  | r^2^=0.1193 | 0.2478 | ns |
| **Fig. 2C (left)** | Corr. DI with exploration behavior:  Novel vs. O2 | 24 | 13 | Spearman correlation & simple linear regression | ctrl | r_s_=0.0757 | 0.7251 | ns |
|  |  |  |  |  |  | r^2^=  3.69e-007 | 0.9978 | ns |
|  |  |  |  |  | tg | r_s_=-0.6703 | 0.0147 | * |
|  |  |  |  |  |  | r^2^=0.3681 | 0.0279 | * |
| **Fig. 2C (right)** | Corr. DI with exploration behavior:  Novel vs. updated | 24 | 13 | Spearman correlation & simple linear regression | ctrl | r_s_=-0.1852 | 0.3862 | ns |
|  |  |  |  |  |  | r^2^=0.0244 | 0.4661 | ns |
|  |  |  |  |  | tg | r_s_=-0.5385 | 0.0611 | ns |
|  |  |  |  |  |  | r^2^=0.2178 | 0.0334 | * |

Abbreviations: O1-2, Original 1-2; ctrl, control; tg, transgenic; MW, Mann-Whitney U

**Supplementary Table 8.** Statistics for memory performance (DI): males and females – Fig. 3

| **Fig. panel** | **Graph description** | **n**  **ctrl M** | **n**  **ctrl F** | **n tg M** | **n tg F** | **Statistical**  **test** | **Stat. metrics** | | **P value** | **Adjusted P value** | **Sig.** |
| --- | --- | --- | --- | --- | --- | --- | --- | --- | --- | --- | --- |
| **Fig. 3B** | Object displacement: males vs. females | 13 | 17 | 14 | 14 | MW  (ctrl vs. tg) with  HB post-hoc | M | MW-U: 54 | 0.0735 | 0.1470 | ns |
|  |  |  |  |  |  |  | F | MW-U=75.5 | 0.0858 | 0.1470 | ns |
|  |  |  |  |  |  | One sample Wilcoxon test (theoretical mean: 0)  with  HB post-hoc | ctrl M | W=89 | 0.0005 | 0.0006 | *** |
|  |  |  |  |  |  |  | ctrl F | W=139.0 | 0.0003 | 0.0006 | *** |
|  |  |  |  |  |  |  | tg M | W=37 | 0.2670 | 0.3452 | ns |
|  |  |  |  |  |  |  | tg F | W=45.0 | 0.1726 | 0.3452 | ns |
| **Fig. 3D**  **(left)** | Updating performance: males | 11 | | 8 | | Two-way ANOVA  with Tukey’s post-hoc | Memory type | F (1.539, 26.17) = 6.302 | 0.0096 | Control:  Novel-O1 vs. Novel-updated  0.0077 | ** |
|  |  |  |  |  |  |  |  |  |  | APP/PS1:  Novel-O1 vs. Novel-updated  0.0318 | * |
|  |  |  |  |  |  |  | GT | F (1, 17) = 2.087 | 0.1667 | - | ns |
|  |  |  |  |  |  | One sample Wilcoxon test (theoretical mean: 0)  with  HB post-hoc | ctrl O1 | W=52 | 0.0186 | 0.0372 | * |
|  |  |  |  |  |  |  | tg O1 | W=20 | 0.1953 | 0.5859 | ns |
|  |  |  |  |  |  |  | ctrl O2 | W=58 | 0.0068 | 0.0204 | * |
|  |  |  |  |  |  |  | tg O2 | W=0 | >0.9999 | >0.9999 | ns |
|  |  |  |  |  |  |  | ctrl updated | W=14 | 0.5771 | 0.5771 | ns |
|  |  |  |  |  |  |  | tg updated | W=-14 | 0.3828 | 0.7656 | ns |
| **Fig. 3D**  **(right)** | Updating performance: females | 13 | | 5 | | Two-way ANOVA  with Tukey’s correction | Memory type | F (1.402, 22.44) = 3.153 | 0.0772 | - | ns |
|  |  |  |  |  |  |  | GT | F (1, 16) = 0.4150 | 0.5286 | - | ns |
|  |  |  |  |  |  | One sample Wilcoxon test (theoretical mean: 0)  with  HB post-hoc | ctrl O1 | W=73 | 0.0081 | 0.0243 | * |
|  |  |  |  |  |  |  | tg O1 | W=11 | 0.1875 | 0.5625 | ns |
|  |  |  |  |  |  |  | ctrl O2 | W=69 | 0.0134 | 0.0268 | * |
|  |  |  |  |  |  |  | tg O2 | W=1 | >0.9999 | >0.9999 | ns |
|  |  |  |  |  |  |  | ctrl updated | W=29 | 0.3396 | 0.3396 | ns |
|  |  |  |  |  |  |  | tg updated | W=1 | >0.9999 | >0.9999 | ns |
| **Fig. 3E**  **(left)** | Updating performance: males  (updated vs. O1) | 11 | | 8 | | Wilcoxon matched-pairs signed rank test  with  HB post-hoc | ctrl | W=-60 | 0.0049 | 0.0098 | ** |
|  |  |  |  |  |  |  | tg | W=-6.0 | 0.7422 | 0.7422 | ns |
| **Fig. 3E**  **(right)** | Updating performance: females  (updated vs. O1) | 13 | | 5 | | Wilcoxon matched-pairs signed rank test  with  HB post-hoc | ctrl | W=-43.0 | 0.1465 | 0.2930 | ns |
|  |  |  |  |  |  |  | tg | W=-9.0 | 0.3125 | 0.3125 | ns |

Abbreviations: O1-2, Original 1-2; ctrl – control; tg – transgenic; M – male; F – female; HB – Holm-Bonferroni; MW – Mann-Whitney U; GT – genotype

**Supplementary Table 9.** Statistics for memory performance (DI): 4-7 and 8-11 months old mice – Fig. 4

| **Fig. panel** | **Graph descr.** | **n**  **ctrl**  **4-7**  **m** | **n**  **ctrl**  **8-11**  **m** | **n tg**  **4-7**  **m** | **n tg**  **8-11**  **m** | **Statistical**  **test** | **Stat. metrics** | | **P value** | **Adjusted P value** | | **Sig.** |
| --- | --- | --- | --- | --- | --- | --- | --- | --- | --- | --- | --- | --- |
| **Fig. 4B** | Object displac. 4-7m and.  8-11m | 11 | 19 | 9 | 19 | MW  (ctrl vs. tg) | 4-7 | MW-U=40 | 0.5027 | 0.5027 | | ns |
|  |  |  |  |  |  |  | 8-11 | MW-U=97 | 0.0140 | 0.0280 | | * |
|  |  |  |  |  |  | One sample Wilcoxon test (theoretical mean: 0) with HB post-hoc | ctrl  4-7 | W=54 | 0.0137 | 0.0411 | | * |
|  |  |  |  |  |  |  | ctrl  8-11 | W=190 | <0.0001 | 0.0004 | | *** |
|  |  |  |  |  |  |  | tg  4-7 | W=25 | 0.1641 | 0.3282 | | ns |
|  |  |  |  |  |  |  | tg  8-11 | W=62 | 0.2251 | 0.3282 | | ns |
| **Fig. 4C**  **(top)** | Object displac. explor.  latency  4-7m and.  8-11m | 11 | 19 | 9 | 19 | MW  (ctrl vs. tg)  with HB post-hoc | 4-7 | MW-U=44 | 0.7103 | 0.7103 | | ns |
|  |  |  |  |  |  |  | 8-11 | MW-U=95 | 0.0119 | 0.0238 | | * |
| **Fig. 4C (bott-om)** | Updating perform.  explor.  latency  4-7m and.  8-11m | 8 | 16 | 4 | 10 | MW  (ctrl vs. tg)  with HB post-hoc | 4-7 | MW-U=11 | 0.4606 | 0.4606 | | ns |
|  |  |  |  |  |  |  | 8-11 | MW-U=54 | 0.1824 | 0.3648 | | ns |
| **Fig. 4E** | Updating perform. 8-11m  (Novel vs. O1, O2 and updated) | 16 | | 10 | | Two-way ANOVA  with Tukey’s correction | Memory type* | F (1.962, 47.09) = 5.731 | 0.0062 | APP/PS1:  Novel-O1 vs. novel-updated  0.0449 | | * |
|  |  |  |  |  |  |  | GT | F (1, 24) = 4.405 | 0.0465 | O1 | 0.9514 | ns |
|  |  |  |  |  |  |  |  |  |  | O2 | 0.0093 | ** |
|  |  |  |  |  |  |  |  |  |  | Upd. | 0.2039 | ns |
|  |  |  |  |  |  | One sample Wilcoxon test (theoretical mean: 0) with HB post-hoc | Ctrl O1 | W=110 | 0.0027 | 0.0054 | | ** |
|  |  |  |  |  |  |  | Tg O1 | W=35 | 0.0820 | 0.2460 | | ns |
|  |  |  |  |  |  |  | Ctrl O2 | W=114 | 0.0017 | 0.0051 | | ** |
|  |  |  |  |  |  |  | Tg O2 | W=-17 | 0.4316 | 0.8632 | | ns |
|  |  |  |  |  |  |  | Ctrl updated | W=56 | 0.1591 | 0.1591 | | ns |
|  |  |  |  |  |  |  | Tg updated | W=-15 | 0.4922 | 0.8632 | | ns |
| **Fig. 4F** | Updating perform.  8-11m  (updated vs. O1) | 16  (paired) | | 10  (paired) | | Wilcoxon matched-pairs signed rank test | ctrl | W=-110 | 0.0027 | 0.0054 | | ** |
|  |  |  |  |  |  |  | tg | W=-7 | 0.7695 | 0.7695 | | ns |

*only significant post-hoc results shown

Abbreviations: O1-2, Original 1-2; ctrl, control; tg, transgenic; HB, Holm-Bonferroni; MW, Mann-Whitney U; GT, genotype; Upd., updated

**Supplementary Table 10.** cFos density quantification in hippocampal and entorhinal cortex regions and their correlation to memory performance – Fig. 5

| **Fig. panel** | **Graph description** | **n**  **(ctrl)** | **n**  **(tg)** | **Statistical**  **test** | **Stat. metrics** | | **P value** | **Sig.** |
| --- | --- | --- | --- | --- | --- | --- | --- | --- |
| **Fig. 5B**  **(top, i-iii)** | cFos+NeuN+ density in hipp. | 16 | 16 | MW  (ctrl vs. tg) | CA1 | MW-U=100 | 0.3045 | ns |
|  |  |  |  |  | CA3 | MW-U=104 | 0.3809 | ns |
|  |  |  |  |  | DG | MW-U=76 | 0.0513 | ns |
| **Fig. 5B**  **(bott-om, iv-vi)** | cFos+NeuN+ density in mEC | 15 | 15 | MW  (ctrl vs. tg) | mEC L2 | MW-U=97 | 0.5393 | ns |
|  |  |  |  |  | mEC L3-4 | MW-U=105 | 0.7748 | ns |
|  |  |  |  |  | mEC L5-6 | MW-U=93 | 0.4363 | ns |
| **Fig. 5D**  **(left)** | cFos+NeuN+ density in CA3 in corr. with memory during object displacement (updated vs. O1) | 14 | 14 | Spearman correlation & simple linear regression | ctrl | r_s_=0.6615 | 0.0121 | * |
|  |  |  |  |  |  | r^2^=0.4208 | 0.0121 | * |
|  |  |  |  |  | tg | r_s_=0.3520 | 0.2160 | ns |
|  |  |  |  |  |  | r^2^=0.1498 | 0.1716 | ns |
| **Fig. 5D**  **(right)** | cFos+NeuN+ density in mEC L3-4 in corr. with memory during object displacement (updated vs. O1) | 14 | 12 | Spearman correlation & simple linear regression | ctrl | r_s_=0.5165 | 0.0615 | ns |
|  |  |  |  |  |  | r^2^=0.1711 | 0.1415 | ns |
|  |  |  |  |  | tg | r_s_=0.4028 | 0.1940 | ns |
|  |  |  |  |  |  | r^2^=0.0896 | 0.3445 | ns |
| **Fig. 5E**  **(left)** | cFos+NeuN+ density in CA1 in corr. with memory during updating performance (novel vs. O2) | 10 | 6 | Spearman correlation & simple linear regression | ctrl | r_s_=-0.6727 | 0.0390 | * |
|  |  |  |  |  |  | r^2^=0.4672 | 0.0293 | * |
|  |  |  |  |  | tg | r_s_=0.0857 | 0.9194 | ns |
|  |  |  |  |  |  | r^2^=0.1653 | 0.4238 | ns |
| **Fig. 5E**  **(right)** | cFos+NeuN+ density in CA1 in corr. with memory during updating performance (novel vs. updated) | 10 | 6 | Spearman correlation & simple linear regression | ctrl | r_s_=0.2371 | 0.5060 | ns |
|  |  |  |  |  |  | r^2^=0.0448 | 0.5570 | ns |
|  |  |  |  |  | tg | r_s_=-0.6571 | 0.1750 | ns |
|  |  |  |  |  |  | r^2^=0.0671 | 0.6201 | ns |

Abbreviations: O1-2, Original 1-2; ctrl, control; tg, transgenic; MW, Mann-Whitney U; CA, Cornu Ammonis; DG, dentate gyrus; mEC, medial entorhinal cortex

**Supplementary Table 11.** Comparison of manual and automated object exploration values – Sup. Fig. 2

| **Mouse ID** | **Session** | **Object** | **Manual time (s)** | **Automatic time**  **(s)** | **Total**  **Time**  **(s)** | **Manual**  **Prop.** | **Automatic Prop.** | **Mean Prop.** | **Diff.**  **Prop.** | **Abs.**  **Diff. Prop.** | **MAE** |
| --- | --- | --- | --- | --- | --- | --- | --- | --- | --- | --- | --- |
| 1 | 1 | 1 | 0.00 | 0.00 | 10.00 | 0.00 | 0.00 | 0.00 | 0.00 | 0.00 | 0.00007 |
| 1 | 1 | 3 | 10.00 | 10.00 | 10.00 | 1.00 | 1.00 | 1.00 | 0.00 | 0.00 |  |
| 1 | 2 | 1 | 3.20 | 6.00 | 20.00 | 0.16 | 0.30 | 0.23 | 0.07 | 0.07 | 0.06 |
| 1 | 2 | 2 | 6.13 | 4.66 | 20.00 | 0.31 | 0.23 | 0.27 | -0.04 | 0.04 |  |
| 1 | 2 | 3 | 5.23 | 2.17 | 20.00 | 0.26 | 0.11 | 0.19 | -0.08 | 0.08 |  |
| 1 | 2 | 4 | 5.43 | 7.13 | 20.00 | 0.27 | 0.36 | 0.31 | 0.04 | 0.04 |  |
| 2 | 1 | 1 | 6.30 | 5.80 | 10.00 | 0.63 | 0.58 | 0.61 | -0.02 | 0.02 | 0.02 |
| 2 | 1 | 3 | 3.70 | 4.17 | 10.00 | 0.37 | 0.42 | 0.39 | 0.02 | 0.02 |  |
| 2 | 2 | 1 | 0.60 | 1.07 | 20.00 | 0.03 | 0.05 | 0.04 | 0.01 | 0.01 | 0.02 |
| 2 | 2 | 2 | 3.33 | 3.40 | 20.00 | 0.17 | 0.17 | 0.17 | 0.00 | 0.00 |  |
| 2 | 2 | 3 | 9.47 | 8.01 | 20.00 | 0.47 | 0.40 | 0.44 | -0.04 | 0.04 |  |
| 2 | 2 | 4 | 6.57 | 7.50 | 20.00 | 0.33 | 0.37 | 0.35 | 0.02 | 0.02 |  |
| 3 | 1 | 1 | 2.23 | 2.93 | 10.00 | 0.22 | 0.29 | 0.26 | 0.04 | 0.04 | 0.04 |
| 3 | 1 | 3 | 7.77 | 7.06 | 10.00 | 0.78 | 0.71 | 0.74 | -0.04 | 0.04 |  |
| 3 | 2 | 1 | 2.77 | 3.90 | 20.00 | 0.14 | 0.20 | 0.17 | 0.03 | 0.03 | 0.04 |
| 3 | 2 | 2 | 4.17 | 2.47 | 20.00 | 0.21 | 0.12 | 0.17 | -0.04 | 0.04 |  |
| 3 | 2 | 3 | 2.43 | 0.90 | 20.00 | 0.12 | 0.05 | 0.08 | -0.04 | 0.04 |  |
| 3 | 2 | 4 | 10.63 | 12.72 | 20.00 | 0.53 | 0.64 | 0.58 | 0.05 | 0.05 |  |
| 4 | 1 | 1 | 1.23 | 1.76 | 10.00 | 0.12 | 0.18 | 0.15 | 0.03 | 0.03 | 0.03 |
| 4 | 1 | 3 | 8.77 | 8.23 | 10.00 | 0.88 | 0.82 | 0.85 | -0.03 | 0.03 |  |
| 4 | 2 | 1 | 1.80 | 0.90 | 20.00 | 0.09 | 0.04 | 0.07 | -0.02 | 0.02 | 0.02 |
| 4 | 2 | 2 | 0.00 | 0.00 | 20.00 | 0.00 | 0.00 | 0.00 | 0.00 | 0.00 |  |
| 4 | 2 | 3 | 13.70 | 12.60 | 20.00 | 0.69 | 0.63 | 0.66 | -0.03 | 0.03 |  |
| 4 | 2 | 4 | 4.50 | 6.50 | 20.00 | 0.23 | 0.32 | 0.27 | 0.05 | 0.05 |  |
| 5 | 1 | 1 | 4.03 | 4.63 | 10.00 | 0.40 | 0.46 | 0.43 | 0.03 | 0.03 | 0.03 |
| 5 | 1 | 3 | 5.97 | 5.34 | 10.00 | 0.60 | 0.53 | 0.57 | -0.03 | 0.03 |  |
| 5 | 2 | 1 | 0.80 | 2.24 | 20.00 | 0.04 | 0.11 | 0.08 | 0.04 | 0.04 | 0.02 |
| 5 | 2 | 2 | 0.60 | 0.23 | 20.00 | 0.03 | 0.01 | 0.02 | -0.01 | 0.01 |  |
| 5 | 2 | 3 | 5.83 | 5.87 | 20.00 | 0.29 | 0.29 | 0.29 | 0.00 | 0.00 |  |
| 5 | 2 | 4 | 12.77 | 11.64 | 20.00 | 0.64 | 0.58 | 0.61 | -0.03 | 0.03 |  |

**Supplementary Table 12.** Statistics for automatic scoring assessment in comparison to manual scoring – Sup. Fig. 2

| **Fig. panel** | **Graph descript-ion** | **n**  **mice** | **n**  **sessions** | **Stat.**  **test** | **Stat. metrics** | **P value** | **Sig.** |
| --- | --- | --- | --- | --- | --- | --- | --- |
| Sup. Fig. 2A | Object exploration proportion manual vs. automatic scoring | 5 | 10 | Spearman correlation & linear regression | r_s_= 0.9509 | <0.0001 | **** |
|  |  |  |  |  | r^2^=0.9341 | <0.0001 | **** |
| Sup. Fig. 2B | Object exploration proportion manual vs. automatic scoring  -  Descriptive statistics | 5 | 10 | Bland-Altman | bias: -0.0003204 | - | - |
|  |  |  |  |  | SD of bias: 0.07012 |  |  |
|  |  |  |  |  | 95% Limits of Agreement:  From:  -0.1377 to 0.1371 |  |  |
| Sup. Fig. 2C | Mean absolute error per session per mouse  -  Descriptive statistics | - | 10 | Mean Absolute Rrror | Min: 7.072e-005 | - | - |
|  |  |  |  |  | Max: 0.05643 |  |  |
|  |  |  |  |  | Range:  0.05636 |  |  |
|  |  |  |  |  | Mean: 0.02757 |  |  |
|  |  |  |  |  | Std. Deviation:  0.01493 |  |  |
|  |  |  |  |  | Std. Error of Mean:  0.004722 |  |  |

**Supplementary Table 13.** Statistics for memory performance (DI) during updating performance without exclusion (pooled) Sup. Fig. 4

| **Fig. panel** | **Graph description** | **n**  **(ctrl)** | **n**  **(tg)** | **Statistical**  **test** | **Stat. metrics** | | **P value** | **Adjusted P value** | **Sig.** |
| --- | --- | --- | --- | --- | --- | --- | --- | --- | --- |
| **Sup. Fig. 4A** | Updating performance (novel vs. O1, O2, updated) | 29 | 22 | One sample Wilcoxon test (theoretical mean: 0) with HB post-hoc | O1 | ctrl: W = 337 | <0.0001 | 0.0003 | *** |
|  |  |  |  |  |  | tg: W = 151 | 0.0127 | 0.0381 | * |
|  |  |  |  |  | O2 | ctrl: W =369 | <0.0001 | 0.0003 | *** |
|  |  |  |  |  |  | tg: W = 99 | 0.1129 | 0.2258 | ns |
|  |  |  |  |  | updated | ctrl: W = 139 | 0.1373 | 0.1373 | ns |
|  |  |  |  |  |  | tg: W = 79 | 0.2072 | 0.2258 | ns |
|  |  |  |  | Two-way ANOVA  with Tukey’s correction | Memory type* | F (1.883, 92.25) = 3.675 | 0.0316 | Control:  Novel-O1 vs. novel-updated  0.0216 | * |
|  |  |  |  |  | GT | F (1, 49) = 0.7805 | 0.3813 | - | ns |
| **Sup. Fig. 4B** | Updating performance (updated vs. O1) | 29 | 22 | Wilcoxon matched-pairs signed rank test | ctrl | W= -199 | 0.0308 | - | * |
|  |  |  |  |  | tg | W=-85 | 0.1762 | - | ns |
| **Sup. Fig. 4C** | Exploration latency: updating perform. | 29 | 22 | MW  (ctrl vs. tg) | MW-U=215 | | 0.0484 | - | * |

*only significant post-hoc results shown

Abbreviations: O1-2, Original 1-2; ctrl, control; tg, transgenic; HB, Holm-Bonferroni; MW, Mann-Whitney U; GT, genotype

**Supplementary Table 14.** Statistics for memory performance during updating performance (males and females) without exclusion (pooled) – Sup. Fig.5

| **Fig. panel** | **Graph description** | **n**  **ctrl** | **n**  **tg** | **Statistical**  **test** | **Stat. metrics** | | **P value** | **Adjusted P value** | **Sig.** |
| --- | --- | --- | --- | --- | --- | --- | --- | --- | --- |
| **Sup.**  **Fig. 5A**  **(left)** | Updating performance: males | 12 | 12 | Two-way ANOVA  with Tukey’s post-hoc | Memory type | F (1.999, 43.97) = 1.442 | 0.2474 | - | ns |
|  |  |  |  |  | GT | F (1, 22) = 0.4771 | 0.4970 | - | ns |
|  |  |  |  | One sample Wilcoxon test (theoretical mean: 0)  with  HB post-hoc | ctrl O1 | W=48 | 0.0640 | 0.1280 | ns |
|  |  |  |  |  | tg O1 | W=34 | 0.2036 | 0.6108 | ns |
|  |  |  |  |  | ctrl O2 | W=62 | 0.0122 | 0.0366 | * |
|  |  |  |  |  | tg O2 | W=12 | 0.6772 | 0.6788 | ns |
|  |  |  |  |  | ctrl updated | W=12 | 0.6772 | 0.6772 | ns |
|  |  |  |  |  | tg updated | W=26 | 0.3394 | 0.6788 | ns |
| **Sup.**  **Fig. 5A**  **(right)** | Updating performance: females | 17 | 10 | Two-way ANOVA  with Tukey’s correction | Memory type | F (1.484, 37.10) = 2.977 | 0.0772 | - | ns |
|  |  |  |  |  | GT | F (1, 25) = 0.1212 | 0.7307 | - | ns |
|  |  |  |  | One sample Wilcoxon test (theoretical mean: 0)  with  HB post-hoc | ctrl O1 | W=131 | 0.0008 | 0.0024 | ** |
|  |  |  |  |  | tg O1 | W=49 | 0.0098 | 0.0294 | * |
|  |  |  |  |  | ctrl O2 | W=129 | 0.0011 | 0.0024 | ** |
|  |  |  |  |  | tg O2 | W=29 | 0.1602 | 0.3204 | ns |
|  |  |  |  |  | ctrl updated | W=67 | 0.1202 | 0.1202 | ns |
|  |  |  |  |  | tg updated | W=17 | 0.4316 | 0.4316 | ns |
| **Sup.**  **Fig. 5B**  **(left)** | Updating performance: males  (updated vs. O1) | 12 | 12 | Wilcoxon matched-pairs signed rank test  with  HB post-hoc | ctrl | W=-72 | 0.0024 | 0.0048 | ** |
|  |  |  |  |  | tg | W=-30 | 0.2661 | 0.2661 | ns |
| **Sup.**  **Fig. 5B**  **(right)** | Updating performance: females  (updated vs. O1) | 17 | 10 | Wilcoxon matched-pairs signed rank test  with  HB post-hoc | ctrl | W=-27 | 0.5477 | 0.8632 | ns |
|  |  |  |  |  | tg | W=-17 | 0.4316 | 0.8632 | ns |

Abbreviations: O1-2, Original 1-2; ctrl, control; tg, transgenic; HB, Holm-Bonferroni; MW, Mann-Whitney U; GT, genotype

**Supplementary Table 15.** Statistics for memory performance during updating performance (4-7 and 8-11 months) without exclusion (pooled) – Sup. Fig. 6

| **Fig. panel** | **Graph descr.** | **n**  **ctrl** | | **n**  **tg** | | **Statistical**  **test** | **Stat. metrics** | | **P value** | **Adjusted P value** | | **Sig.** |
| --- | --- | --- | --- | --- | --- | --- | --- | --- | --- | --- | --- | --- |
| **Sup.**  **Fig. 6A** | Object perform.  explor.  latency  4-7m and.  8-11m | **4-7**  **m** | **8-11**  **m** | **4-7**  **m** | **8-11**  **m** | MW  (ctrl vs. tg)  with HB post-hoc | 4-7 | MW-U=43 | 0.6556 | 0.6556 | | ns |
|  |  | 11 | 18 | 9 | 16 |  | 8-11 | MW-U=82 | 0.0326 | 0.0652 | | ns |
| **Sup.**  **Fig. 6B** | Updating perform. 8-11m  (Novel vs. O1, O2 and updated) | 18 | | 16 | | Two-way ANOVA  with Tukey’s correction | Memory type | F (1.810, 57.91) = 2.643 | 0.0849 | - | | ns |
|  |  |  |  |  |  |  | GT | F (1, 32) = 5.530 | 0.0250 | O1 | 0.3995 | ns |
|  |  |  |  |  |  |  |  |  |  | O2 | 0.0053 | ** |
|  |  |  |  |  |  |  |  |  |  | Upd. | 0.3584 | ns |
|  |  |  |  |  |  | One sample Wilcoxon test (theoretical mean: 0) with HB post-hoc | Ctrl O1 | W=114 | 0.0010 | 0.0020 | | ** |
|  |  |  |  |  |  |  | Tg O1 | W=58 | 0.1400 | 0.4200 | | ns |
|  |  |  |  |  |  |  | Ctrl O2 | W=149 | 0.0004 | 0.0012 | | ** |
|  |  |  |  |  |  |  | Tg O2 | W=-28 | 0.4866 | 0.9732 | | ns |
|  |  |  |  |  |  |  | Ctrl updated | W=79 | 0.0898 | 0.0898 | | ns |
|  |  |  |  |  |  |  | Tg updated | W=1 | 0.9899 | 0.9899 | | ns |
| **Sup.**  **Fig. 6C** | Updating perform.  8-11m  (updated vs. O1) | 18  (paired) | | 16  (paired) | | Wilcoxon matched-pairs signed rank test | ctrl | W=-131 | 0.0028 | 0.0056 | | ** |
|  |  |  |  |  |  |  | tg | W=-14 | 0.7197 | 0.7197 | | ns |

Abbreviations: O1-2, Original 1-2; ctrl, control; tg, transgenic; HB, Holm-Bonferroni; MW, Mann-Whitney U; GT, genotype; Upd., updated

**Supplementary Table 16.** Exploration parameters during OUL paradigm – Supplementary Fig. 7

| **Fig. panel** | **Graph description** | **n**  **(ctrl)** | **n**  **(tg)** | **Statistical**  **test** | **Stat. metrics** | | **P value** | **Sig.** |
| --- | --- | --- | --- | --- | --- | --- | --- | --- |
| **Sup. Fig. 7A (left)** | Training:  Total displace-ment | 35 | 33 | Two-way ANOVA with Bonferroni’s correction | genotype | F (1, 66) = 0.007260 | 0.9324 | ns |
| **Sup. Fig. 7A (right)** | Training:  Median speed | 35 | 33 | Two-way ANOVA with Bonferroni’s correction | genotype | F (1, 66) = 0.1195 | 0.7307 | ns |
| **Sup. Fig. 7B (left)** | Object  displacem-ment:  Total displacem-ment | 30 | 28 | MW  (ctrl vs. tg) | MW-U=320 | | 0.1222 | ns |
| **Sup. Fig. 7B (right)** | Updating perform.:  Total  displacem-ment | 24 | 13 | MW  (ctrl vs. tg) | MW-U=138 | | 0.5825 | ns |
| **Sup. Fig. 7C (left)** | Object  displacem-ment:  Median speed | 30 | 28 | MW  (ctrl vs. tg) | MW-U=372 | | 0.4627 | ns |
| **Sup. Fig. 7C (right)** | Updating perform.:  Median speed | 24 | 13 | MW  (ctrl vs. tg) | MW-U=149 | | 0.8384 | ns |

Abbreviations: ctrl, control; tg, transgenic; MW, Mann-Whitney U

**Supplementary Table 17.** NeuN+cFos+ vs. NeuN-cFos+ cell distribution in hippocampus and medial entorhinal cortex layers – Supplementary Fig. 8 – 10

| **Fig. panel** | **Graph description** | **n**  **(ctrl)** | **n**  **(tg)** | **Statistical**  **test** | **Stat. metrics** | | **P value** | **Holm-Bonfe-rroni**  **correc.** | **Sig.** |
| --- | --- | --- | --- | --- | --- | --- | --- | --- | --- |
| **Sup. Fig.**  **8C** | NeuN+  cFos+  vs.  NeuN-  cFos+  distr.  CA1 & CA3 | 16 | 16 | MW  (ctrl vs. tg) | CA1 | MW-U=104.5 | 0.2757 | >0.9999 | ns |
|  |  |  |  |  | CA3 | MW-U=123 | 0.8830 | >0.9999 | ns |
| **Sup. Fig. 9B** | NeuN+  cFos+  vs.  NeuN-  cFos+  distr.  DG | 16 | 16 | MW  (ctrl vs. tg) | MW-U=92 | | 0.1312 | 0.7872 | ns |
| **Sup. Fig. 10B**  **(top)** | NeuN+  cFos+  vs.  NeuN-  cFos+  distr.  mEC L2 | 15 | 15 | MW  (ctrl vs. tg) | MW-U=97.5 | | 0.4828 | >0.9999 | ns |
| **Sup. Fig. 10B**  **(mid)** | NeuN+  cFos+  vs.  NeuN-  cFos+  distr.  mEC L3-4 | 14 | 15 | MW  (ctrl vs. tg) | MW-U=98 | | 0.7414 | >0.9999 | ns |
| **Sup. Fig. 10B**  **(bott-om)** | NeuN+  cFos+  vs.  NeuN-  cFos+  distr.  mEC L5-6 | 15 | 15 | MW  (ctrl vs. tg) | MW-U=90 | | 0.2241 | >0.9999 | ns |

Abbreviations: O1-2, Original 1-2; ctrl, control; tg, transgenic; MW, Mann-Whitney U; CA, Cornu Ammonis; DG, dentate gyrus; mEC, medial entorhinal cortex

**Supplementary Table 18.** Sex- and age-dependent NeuN+cFos+ cell distribution in hippocampus and medial entorhinal cortex layers – Supplementary Fig. 11

| **Fig. panel** | **Graph description** | **n**  **(ctrl)** | | **n**  **(tg)** | | **Statistical**  **test** | **Stat. metrics** | | **P value** | **Sig.** |
| --- | --- | --- | --- | --- | --- | --- | --- | --- | --- | --- |
|  |  | **HC** | **EC** | **HC** | **EC** |  |  |  |  |  |
| **Sup. Fig. 11A**  **(left)** | cFos+NeuN+ density in hipp. and mEC:  males | 5 | 5 | 8 | 7 | MW  (ctrl vs. tg) | CA1 | MW-U=20 | >0.9999 | ns |
|  |  |  |  |  |  |  | CA3 | MW-U=17 | 0.7242 | ns |
|  |  |  |  |  |  |  | DG | MW-U=19 | 0.9433 | ns |
|  |  |  |  |  |  |  | mEC L2 | MW-U=16 | 0.8763 | ns |
|  |  |  |  |  |  |  | mEC L3-4 | MW-U=11 | 0.3434 | ns |
|  |  |  |  |  |  |  | mEC L5-6 | MW-U=7 | 0.1061 | ns |
| **Sup. Fig. 11A**  **(right)** | cFos+NeuN+ density in hipp. and mEC:  females | 11 | 10 | 8 | 8 | MW  (ctrl vs. tg) | CA1 | MW-U=22 | 0.0754 | ns |
|  |  |  |  |  |  |  | CA3 | MW-U=31 | 0.3100 | ns |
|  |  |  |  |  |  |  | DG | MW-U=10 | 0.0036 | ** |
|  |  |  |  |  |  |  | mEC L2 | MW-U=36 | 0.7618 | ns |
|  |  |  |  |  |  |  | mEC L3-4 | MW-U=39 | 0.9654 | ns |
|  |  |  |  |  |  |  | mEC L5-6 | MW-U=40 | >0.9999 | ns |
| **Sup. Fig. 11B**  **(left)** | cFos+NeuN+ density in hipp. and mEC:  4 – 7 months | 9 | 8 | 8 | 8 | MW  (ctrl vs. tg) | CA1 | MW-U=32 | 0.7430 | ns |
|  |  |  |  |  |  |  | CA3 | MW-U=34 | 0.8884 | ns |
|  |  |  |  |  |  |  | DG | MW-U=24 | 0.2766 | ns |
|  |  |  |  |  |  |  | mEC L2 | MW-U=13 | 0.0499 | * |
|  |  |  |  |  |  |  | mEC L3-4 | MW-U=30 | 0.8785 | ns |
|  |  |  |  |  |  |  | mEC L5-6 | MW-U=28 | 0.7209 | ns |
| **Sup. Fig. 11B**  **(right)** | cFos+NeuN+ density in hipp. and mEC:  8 – 9 months | 7 | 7 | 8 | 7 | MW  (ctrl vs. tg) | CA1 | MW-U=17 | 0.2319 | ns |
|  |  |  |  |  |  |  | CA3 | MW-U=13 | 0.0939 | ns |
|  |  |  |  |  |  |  | DG | MW-U=11 | 0.0541 | ns |
|  |  |  |  |  |  |  | mEC L2 | MW-U=20 | 0.6200 | ns |
|  |  |  |  |  |  |  | mEC L3-4 | MW-U=19 | 0.5350 | ns |
|  |  |  |  |  |  |  | mEC L5-6 | MW-U=19 | 0.5350 | ns |

Abbreviations: HC, hippocampus; (m)EC, (medial) entorhinal cortex; ctrl, control; tg, transgenic; MW, Mann-Whitney U
